# Towards precision oncology: a multi-level cancer classification system integrating liquid biopsy and machine learning

**DOI:** 10.1186/s13040-025-00439-8

**Published:** 2025-04-11

**Authors:** Amr Eledkawy, Taher Hamza, Sara El-Metwally

**Affiliations:** 1 Department of Computer Science, Faculty of Computers and Information, Mansoura University, P.O. Box: 35516, Mansoura, Egypt; 2 Biomedical Informatics Department, Faculty of Computer Science and Engineering, New Mansoura University, Gamasa, 35712 Egypt

**Keywords:** Multi-cancer classification, Majority vote feature selection, Ensemble learning, Liquid biopsy, CfDNA/ctDNA, Protein biomarkers

## Abstract

**Background:**

Millions of people die from cancer every year. Early cancer detection is crucial for ensuring higher survival rates, as it provides an opportunity for timely medical interventions. This paper proposes a multi-level cancer classification system that uses plasma cfDNA/ctDNA mutations and protein biomarkers to identify seven distinct cancer types: colorectal, breast, upper gastrointestinal, lung, pancreas, ovarian, and liver.

**Results:**

The proposed system employs a multi-stage binary classification framework where each stage is customized for a specific cancer type. A majority vote feature selection process is employed by combining six feature selectors: Information Value, Chi-Square, Random Forest Feature Importance, Extra Tree Feature Importance, Recursive Feature Elimination, and L1 Regularization. Following the feature selection process, classifiers—including eXtreme Gradient Boosting, Random Forest, Extra Tree, and Quadratic Discriminant Analysis—are customized for each cancer type individually or in an ensemble soft voting setup to optimize predictive accuracy. The proposed system outperformed previously published results, achieving an AUC of 98.2% and an accuracy of 96.21%. To ensure reproducibility of the results, the trained models and the dataset used in this study are made publicly available via the GitHub repository (https://github.com/SaraEl-Metwally/Towards-Precision-Oncology).

**Conclusion:**

The identified biomarkers enhance the interpretability of the diagnosis, facilitating more informed decision-making. The system's performance underscores its effectiveness in tissue localization, contributing to improved patient outcomes through timely medical interventions.

**Supplementary Information:**

The online version contains supplementary material available at 10.1186/s13040-025-00439-8.

## Introduction

Cancer costs millions of lives each year and is one of the leading causes of death globally, accounting for ten million fatalities yearly [1]. Unfortunately, the number of cancer-related fatalities is predicted to increase even in developed countries [2]. Surgery alone can often effectively treat localized cancers without systemic therapy [3]. Surgical excision is seldom curative if distant metastases have taken place. Therefore, there is a significant emphasis on research methods for early cancer detection before it spreads to distant sites. The early detection of cancer enables timely medical interventions, leading to a reduction in patient mortality rates and better outcomes for a wide range of cancer types [4].

In recent years, various methods have increased the accessibility of early cancer detection [5]. In particular, a blood test process called liquid biopsy has become a tool for early cancer identification. The test depends on mutations and genetic changes found in circulating tumor DNA (ctDNA), circulating cell-free DNA (cfDNA), or molecular biomarkers [6]. The principle that mutant plasma DNA templates come from dying cancer cells is the foundation of liquid biopsy, offering highly specific markers for identifying neoplasia. RNA strands, DNA mutations, proteins, and protein fragments are typical molecular biomarkers that can provide valuable insights into disease prognosis in patients [7].

A multi-analytic testing strategy that evaluates mutations in somatic variants within cfDNA, ctDNA, and a range of protein biomarkers from blood plasma can improve early cancer detection by employing different Machine Learning (ML) techniques [8]. ML enables cancer detection employing a range of data forms, including liquid biopsy, clinical, and pathological data [6, 9, 10]. Integrating this valuable marker information into advancements in artificial intelligence has made it possible to develop accurate tools for early cancer prediction.

This paper introduces a system designed to identify seven specific types of cancer: colorectal, breast, upper gastrointestinal (GI), lung, pancreas, ovarian, and liver cancer. The system begins by collecting liquid biopsy blood samples and analyzing protein biomarker concentrations and plasma cfDNA/ctDNA mutations obtained from healthy controls and cancer patients. The proposed approach employs a multi-level binary classification system, creating seven distinct datasets, each designed to target a specific type of cancer among other malignancies. This process involves a comprehensive feature selection procedure that integrates Information Value (IV), Chi-Square, Random Forest (RF) feature importance, Extra Tree (ET) feature importance, Recursive Feature Elimination (RFE), and L1 regularization techniques. Following reduction, the datasets are subjected to model training using algorithms tailored to the specific cancer types. These algorithms include eXtreme Gradient Boosting (XGBoost), RF, ET, and Quadratic Discriminant Analysis (QDA), which are applied individually or in ensemble soft voting configurations.

The main contribution of the paper can be summarized as follows:

- The proposed system employs liquid biopsy to offer a non-invasive method for detecting seven distinct types of cancer at an early stage.
- Employing a voting technique, the system performs comprehensive feature selection by incorporating six methods: IV, Chi-Square, RF, ET, RFE, and L1 regularization. This approach aims to identify the most important features, thereby improving model interpretability.
- The system utilized various machine learning classifiers to customize the modeling process for each cancer type's specific characteristics. Through ensemble soft voting configurations, it leverages the strengths of classifiers such as XGBoost, RF, ET, and QDA, individually or collectively, ensuring accurate predictions.
- The system's evaluation results reached an Area Under the Curve (AUC) of 98.2% and an average accuracy of 96.21%, highlighting the system's capacity to enhance clinical follow-up protocols.

The structure of the paper is as follows: The "Related Work" section provides an overview of previously reported approaches that have used liquid biopsy for the early detection of cancer. In the "Materials and Methods" section, we present the proposed framework for multi-cancer detection, integrating liquid biopsy and ML techniques, along with details about the dataset used in the training process. The "Experimental Results" section presents the outcomes obtained at various stages of the proposed methodology and includes comparative analyses with previously published approaches to establish benchmarking performance. The "Discussion" section comprehensively evaluates the proposed system, highlighting its robustness, offering clinically interpretable insights, and identifying its limitations. Finally, the "Conclusion" section summarizes the study's key findings. It explores potential future research directions in multi-cancer diagnosis, focusing on integrating biological biomarkers and artificial intelligence techniques.

## Related work

Machine learning has been increasingly applied to cancer diagnostics, covering a wide range of cancer types, including colorectal [11], thyroid [12], lung [13], breast [14], and brain [15] tumors, demonstrating its potential in improving early detection and classification accuracy. Many studies have explored cancer prediction and classification using machine learning techniques, such as pACP-HybDeep [16], iACP-GAEnsC [17], cACP-2LFS [18], and cACP-DeepGram [19], which leverage deep learning, ensemble learning, and feature selection methods to enhance predictive accuracy and robustness.

Scholars are now investigating somatic changes associated with cancer in cfDNA as a non-invasive method for early cancer diagnosis [8, 20, 21], such as gastric [22], colorectal [23], lung [24], and breast [25]. Using deep learning methods [26], conjunctive Bayesian networks [27], and network-based multi-task learning models [28] with liquid biopsy data designed to predict cancer is advancing the field of cancer research.

Cohen et al. [8] conducted a comprehensive data collection effort from patients diagnosed with nonmetastatic cancers affecting various organs, including the ovary, liver, stomach, pancreas, esophagus, colorectum, lung, and breast. This dataset includes features of concentrations of protein biomarkers, mutations detected in plasma cfDNA/ctDNA, and important clinical characteristics such as ethnicity, sex, age, and histology. Specifically, the dataset has measurements for 39 distinct protein biomarkers present in plasma samples and an omega score computed from mutations identified in the samples. Their approach, CancerSEEK, was employed to classify seven different cancer types, with esophageal and gastric cancers grouped for analysis. Using a random forest classifier and a tenfold cross-validation technique, they achieved a classification accuracy of 62.32% in their experiments.

Wong et al. [9] introduced a cancer localization framework called CancerA1DE, which relies on Aggregating One-Dependence Estimators (A1DE). They used the Cohen et al. dataset and applied the minimum description length principle to discretize continuous marker features. They classified seven distinct cancer types using the omega score, gender, and 39 protein biomarkers.

Rahaman et al. [6] introduced CancerEMC, a cancer localization system utilizing a Bagging Ensemble Meta Classifier on the Cohen et al. dataset. The Synthetic Minority Oversampling Technique (SMOTE) is used to resolve the dataset imbalance problem, and the Random Forest (RF) is implemented as a feature selection technique. Multiple experiments with different sample sizes of data were conducted in their study. When using 626 cancer patients to detect seven cancer types, they achieved 74.12% accuracy using omega score, gender, and 19 biomarker features selected through RF on the data before applying SMOTE. After applying SMOTE, the system achieved 91.5% accuracy. They detected cancer types in 1,817 people with 83.49% accuracy before SMOTE and 95.98% after. Cancer localization accuracy was 74.22% before SMOTE and 93.98% after SMOTE for 1,005 cancer patients. All experiments use tenfold cross-validation.

Halner et al. [10] proposed the DEcancer framework for early cancer detection on the Cohen et al. dataset. The framework started by partitioning the dataset into a 20% test set, while the remaining data was used for training and validation in a 200-fold Monte Carlo cross-validation configuration. They applied various data augmentation techniques to the training data and optimized the classifier model through feature selection and hyperparameter tuning during fold validation. Using the independent t-test, they compared the performance of the classifier models with different feature sets, ensuring no statistically significant performance difference. The best data processing framework, classifier models, and feature set were selected. They then retrained the models using all combined training and validation data and evaluated them on the test set. With 39 biomarker characteristics, omega score, age, sex, and ethnicity data, their framework obtained 91.88% average AUC for cancer localization with 1005 cancer patients and 94.13% with 1817 individuals.

While CancerA1DE, CancerSEEK, CancerEMC, and DEcancer can help in early cancer detection by applying ML techniques on liquid biopsy data, CancerSEEK and CancerA1DE exhibit low accuracy in cancer-type localization. Furthermore, the applicability of the DEcancer framework is hindered by the lack of information regarding the classifiers and feature selectors utilized in their study. A summary of the related work can be found in Table 1.

**Table 1:** Comparing multi-cancer classification models using the Cohen et al. dataset

Study	#features	#samples	AUC	Accuracy
Cohen et al., 2018 [8]	41	626	91%	62.32**%**
Wong et al., 2019 [9]	41	626	92.1%	69.64%
Rahaman et al., 2021 [6]	21	626	93.8%	74.12**%**
	41	1005	N/A	74.29%
	43	1817	98%	83.49%
Halner et al., 2023 [10]	43	1005	91.88%	N/A
	43	1817	94.13%	

## Materials and methods

**Fig. 1:**
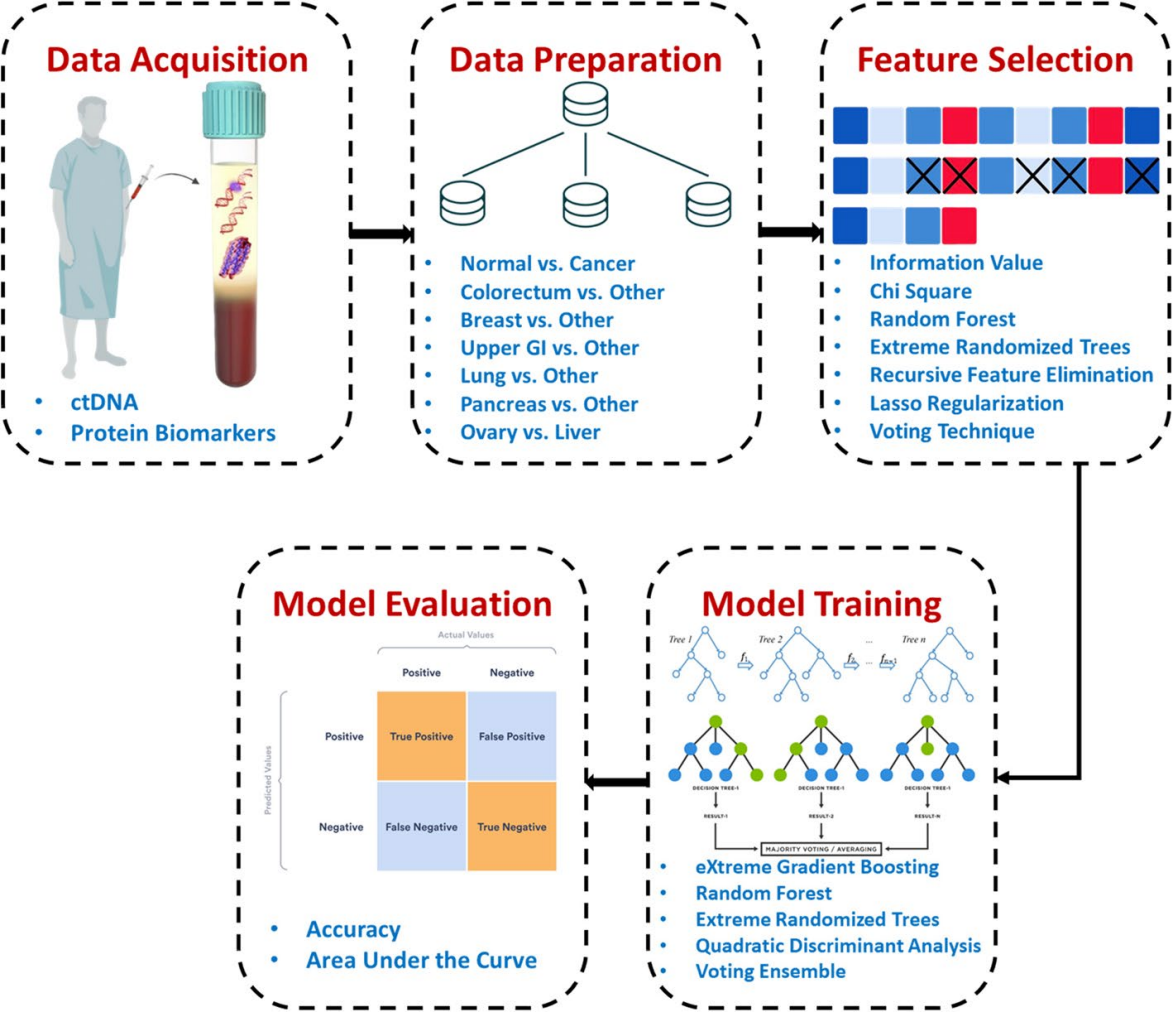
An integrated framework for multi-cancer detection based on liquid biopsy and machine learning

The main goal of this study is to utilize ML techniques on liquid biopsy data to enhance multi-cancer early detection. The proposed approach uses key stages for cancer classification, including data collection, preparation, feature selection, model training, and evaluation (refer to Fig. 1). We utilized the publicly available dataset from Cohen et al. [8], including blood plasma cfDNA/ctDNA mutations, protein biomarker concentrations, and clinical features from cancer patients and healthy controls. The dataset is then prepared for multi-level binary classification, and feature selection methods are used to find the most essential characteristics, which results in a reduced-dimensional dataset for ML classifier training. Finally, the trained machine learning models are evaluated using different performance metrics. Every stage is elaborated upon in the sections that follow.

### Data acquisition

In this study, we utilize the dataset from Cohen et al. [8], which includes blood plasma cfDNA/ctDNA mutations, protein biomarker concentrations, and clinical features from cancer patients and healthy controls. Of the 1817 blood test samples, 1005 were cancer patients diagnosed at age 63 on average. The median age of cancer patients upon diagnosis was 64, ranging from 22 to 93.

**Fig. 2:**
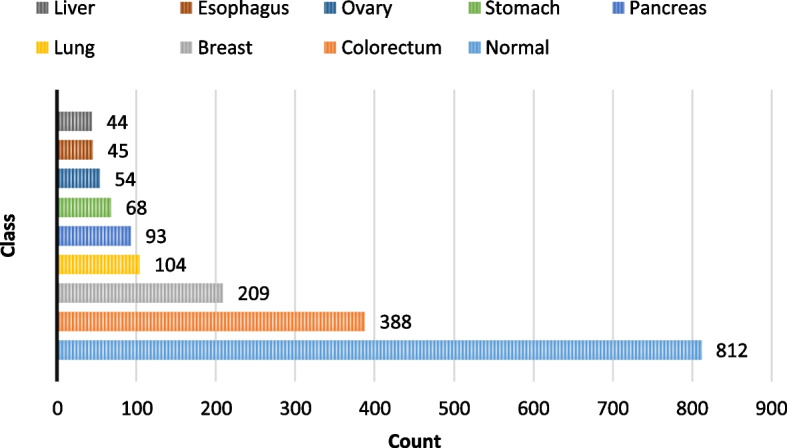
Cohen et al. dataset classes’ distribution

No patients in the dataset had distant metastases nor undergone neoadjuvant chemotherapy before blood sample collection. The dataset consists of cancers from different organs such as breast, lung, colorectum, liver, ovary, stomach, pancreas, and esophagus. Clinical blood diagnostics for early identification of these eight cancers are currently unavailable. In addition, there are 812 healthy controls in the dataset, with an average age of 49 and a median of 55, with a range of 17 to 88 years. They had no malignancy, chronic kidney disease, autoimmune, or high-grade dysplasia history. A summary of the dataset's demographic characteristics is in Table 2. The class distribution in the dataset is illustrated in Fig. 2.

**Table 2:** Demographic characteristics of Cohen et al. dataset

		**Cancer** (1005)	**Normal** (812)
**Sex**	Female	543 (29.9%)	378 (20.8%)
	Male	462 (25.4%)	434 (23.9%)
**Race**	Caucasian	675 (37.1%)	332 (18.27%)
	Asian	301 (16.6%)	22 (1.2%)
	Black	14 (0.8%)	154 (8.48%)
	Hispanic	1 (0.05%)	76 (4.2%)
	Other	14 (0.8%)	228 (12.5%)
**Age** (years) (mean, median)		22- 93 (49, 55)	17- 88 (63, 64)

A total of 39 plasma protein biomarker concentrations are described in Table S1 (Online Resource 1), and an omega score derived from cfDNA mutations is included in the dataset. The Mutant Allele Frequency (MAF) in four plasma cfDNA Unique Identifier sequences (UIDs) determines the omega score. This score is computed according to Eq. (1).

1 $$\Omega =\sum_{i=1}^{w}{w}_{i}\times \mathit{ln}\frac{{p}_{i}^{C}}{{p}_{i}^{N}}$$

where $w$ is the number of wells in cfDNA, ${w}_{i}$ is the ratio of ${UIDs}_{i}$ (the number of Unique Identifiers in the ${i}^{th}$ well of plasma sample $s$) to $UIDs$ (the total number of Unique Identifiers), ${p}_{i}^{C}$ denotes the *p*-value of cancer sample $C$ in the MAF distribution of the ${i}^{th}$ well, while ${p}_{i}^{N}$ represents the *p*-value of normal sample $N$ in the MAF distribution of the same well.

Age, sex, histology, and ethnicity are clinical variables incorporated into the dataset. In this study, the histopathology attribute, which provides microscopic characteristics of cancer cells/tissues to differentiate between different types of cancer, is not utilized as an input attribute for cancer identification. The sex attribute aids in the diagnosis of ovarian and breast cancers due to particular traits presented in female patients. Also, ethnicity reflects genetic and physical traits that might affect cancer detection.

Thus, the dataset contains 43 essential features for each individual, ensuring a comprehensive representation of biological and demographic factors. These include 39 biomarker features (Table S1, Online Resource 1), three demographic features (age, sex, and ethnicity), and the omega score, which quantifies cfDNA mutations. Integrating molecular and demographic variables enhances the dataset's robustness, supporting more accurate and generalizable predictive modeling.

### Data preparation

**Fig. 3:**
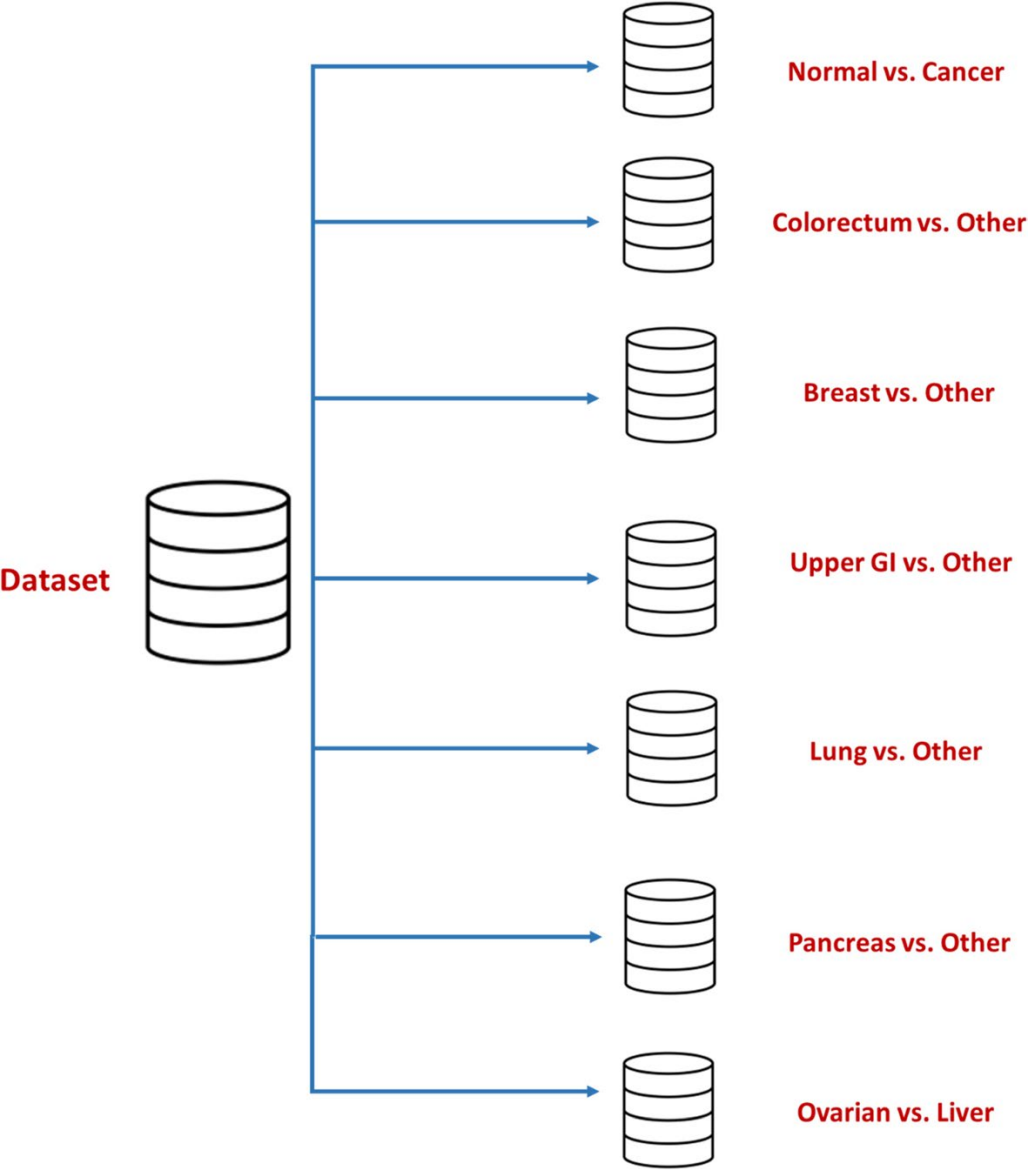
Multi-level binary classification workflow for cancer dataset refinement

The original dataset is compiled to conduct multi-level binary classification into seven datasets, as illustrated in Fig. 3. Esophageal and gastric malignancies are classified as upper gastrointestinal (GI) cancer, based on the suggestion of Cohen et al. [8] study. The dataset is first used for a preliminary binary classification job to discriminate normal and malignant samples. Normal cases are then eliminated from the data. The remaining data creates a binary dataset labeled ‘target cancer’ or ‘other cancers’. This stage aims to separate target cancer patients from other cancers. Iteratively removing the ‘target cancer’ from the data and creating fresh binary classification datasets continues, progressively narrowing down the focus to address other cancer types, as shown in Algorithm 1.

Initially, we transform the original dataset, causing the labels to be categorized into two binary classes: normal or cancerous. Following this, instances labeled as normal are eliminated from the original data. The remaining dataset is then used to construct a new dataset where the labels are categorized as either colorectal or other cancer types. Subsequently, instances corresponding to colorectal cancer are removed from the dataset, and a fresh dataset is established. In this new dataset, the labels are designated as either breast cancer or other types of cancer. This process consistently assigns the class with the highest instances as the first class while combining the remaining classes into the second class. Seven unique datasets are produced from this stage-by-stage refining, each tailored for binary classification and focusing on a particular cancer type compared to the other malignancies.

**Table:**
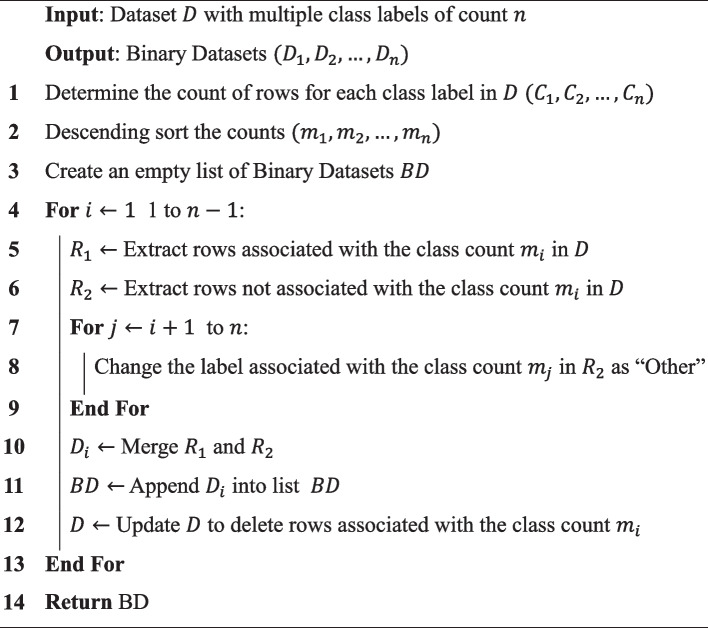
Algorithm 1: Splitting a multi-class dataset into multiple binary datasets

### Feature selection

The feature selection process employs a voting technique that integrates several methods, including IV, Chi-square, RF feature importance, ET feature importance, RFE, and L1 regularization.

IV [29] measures the strength of association between a variable and a binary outcome. By quantifying the extent to which a feature can distinguish between different outcomes, IV provides valuable insights into the variable's relevance for predictive modeling. A higher IV value indicates a stronger discriminatory power. IV is based on the Weight of Evidence (WoE) [29]. WoE works by recording variable values into groups and assigning a unique WoE value to each group to generate the largest difference between the recorded ones. We used monotonic binning [30] in the proposed methodology to create the groups. The primary objective of monotonic binding is to transform a feature by grouping its values into distinct bins while preserving the monotonic relationship with the target variable.

Assume a feature $X$ with $n$ groups $\left\{{x}_{1}, {x}_{2}, \dots , {x}_{n}\right\}$. The WoE of the group $i$ referred to as ${x}_{i}$ is computed from Eq. (2).

2 $$WoE\left({x}_{i}\right)=\text{ln}\left(\frac{C1\left({x}_{i}\right)}{C2\left({x}_{i}\right)}\right)$$

where $C1\left({x}_{i}\right)$ refers to the percentage of positive class in the group ${x}_{i}$, and $C2\left({x}_{i}\right)$ refers to the percentage of negative class in the group ${x}_{i}$. The WoE holds a value of 0 when the distribution of the two classes is equal, indicating that the evaluated variable fails to differentiate between the classes. Utilizing the computed WoE values, we calculate the IV for each feature. The IV for a group $i$ of a feature $X$ with $n$ groups is calculated as in Eq. (3).

3 $$IV\left(X\right)=\sum_{i=1}^{n}\left(C1\left({x}_{i}\right)-C2\left({x}_{i}\right)\right)\times WoE\left({x}_{i}\right)$$

When the IV is below 0.02, the variable is inadequate for distinguishing between the two classes. IV values ranging from 0.02 to 0.1 suggest a weak discriminatory capacity, while those between 0.1 and 0.3 indicate a moderate discriminatory ability. An IV exceeding or equal to 0.3 denotes a strong capacity for discrimination between the two classes.

Chi-square $\chi^2$ [31] is a statistical measure that assesses the independence or association between variables within a dataset. It determines if there is a relationship between two variables by comparing the observed frequency of their co-occurrence to what would be expected under the assumption of independence. In the context of feature selection, $\chi^2$ is used to determine feature relevance to the target class. Features with higher Chi-square values indicate a potential significance in predicting the target outcome. Assume the feature ${X}_{i}$ and the class label ${y}_{j}$, the $\chi^2$ is defined in Eq. (4).

4 $$\chi^2\left(X_i,Y_j\right)=\frac{N\times\left(TZ-YV\right)^2}{\left(T+V\right)\times\left(T+Z\right)\times\left(V+Z\right)\times\left(Y+Z\right)}$$

where $T$ is the frequency of the feature ${X}_{i}$ and class label ${y}_{j}$ in the dataset, $V$ is the frequency of ${X}_{i}$ appearing without ${y}_{j}$, $Y$ is the frequency of ${y}_{j}$ appearing without ${X}_{i}$, $Z$ is the frequency of neither ${y}_{j}$ nor ${X}_{i}$ appearing together in the dataset, $N$ is the total number of records, $i=1, 2, \dots , 43$ features, and $j=1, -1$ class labels.

RF feature importance [32] operates within the context of the RF algorithm, which constructs an ensemble of decision trees. The importance of a feature is determined by observing how much the accuracy of the model changes when that feature is randomly shuffled or perturbed. Features that contribute more to the model's predictive accuracy are assigned higher importance scores.

Each node of the tree within the RF model [33] is constructed using a random feature selection process. RF employs the Out-Of-Bag (OOB) error estimation method, which utilizes a sample set not used in training the current tree. For each feature $X$ operating in tree $j$, RF estimates the OOB error, denoted as $err\left({X}^{j}\right)$, and then randomly exchange the feature’s value with one of its values in the OOB set. Subsequently, the OOB error is re-estimated for the new value of the feature, denoted as $err\left({X}_{oob}^{j}\right)$. The importance score of a specific feature is determined as the mean across $n$ trees of the difference between the OOB error estimations before and after the feature value exchange, as calculated in Eq. (5).

5 $$RF\left({X}^{j}\right)=\frac{1}{n}\sum_{j}\left|err\left({X}^{j}\right)-err\left({X}_{oob}^{j}\right)\right|$$

ET feature importance [34], which depends on the ET classifier, is distinguished from RF's approach of splitting thresholds. While RF employs optimal thresholds based on feature information for splits, ET selects these thresholds at random. The ET method assesses feature importance similarly to RF by perturbing or randomizing specific features and measuring the ensuing decrease in predictive model accuracy.

RFE [35] operates by iteratively identifying and removing less important features from a dataset. RFE begins by training a model on the entire set of features and then ranking them based on their importance scores. The least significant feature is removed, and the model is retrained on the reduced feature set. This process is repeated iteratively, with the least important feature being eliminated in each iteration until a specified number of features is reached, which is 21 in this study. In the proposed methodology, logistic regression is used to rank each feature obtained from the coefficient attribute.

L1 regularization [36] operates by incorporating a penalty term into the model's objective function, which is proportional to the absolute values of the coefficients. This penalty encourages many coefficients to be precisely reduced to zero during optimization, facilitating automated feature selection. Mathematically, L1 regularization integrates a linear model with an additional regularization term. Within the proposed framework, a linear Support Vector Machine (SVM) is employed as the linear model.

Suppose $Y={\left({y}_{1}, {y}_{2}, \dots , {y}_{n}\right)}^{T}$ is the response vector, and ${x}_{j}={\left({x}_{1j}, {x}_{2j}, \dots , {x}_{nj}\right)}^{T}, j=\text{1,2},\dots ,p$ are linearly independent predictors. Define $X=\left[{x}_{1}, {x}_{2},\dots , {x}_{p}\right]$ as the predictor matrix, assuming standardized data, Eq. (6) provides the L1 estimates for the coefficients of a linear model.

6 $$\widehat{\beta }=\underset{\beta }{\text{arg min}}\Vert Y-\sum_{j=}^{p}{x}_{j}{\beta }_{j}\Vert +\lambda \sum_{j=1}^{p}\left|{\beta }_{j}\right|$$

The parameter $\lambda$, the L1 regularization parameter, controls the penalty strength, with higher values leading to increased shrinkage. $\widehat{\beta }$ serves as an unbiased estimate of the degrees of freedom of L1, facilitating the creation of an adaptive model selection metric for optimal L1 fitting. During optimization, some coefficients may shrink to zero $\left({\widehat{\beta }}_{j}=0\right)$, effectively excluding them from the model.

**Fig. 4:**
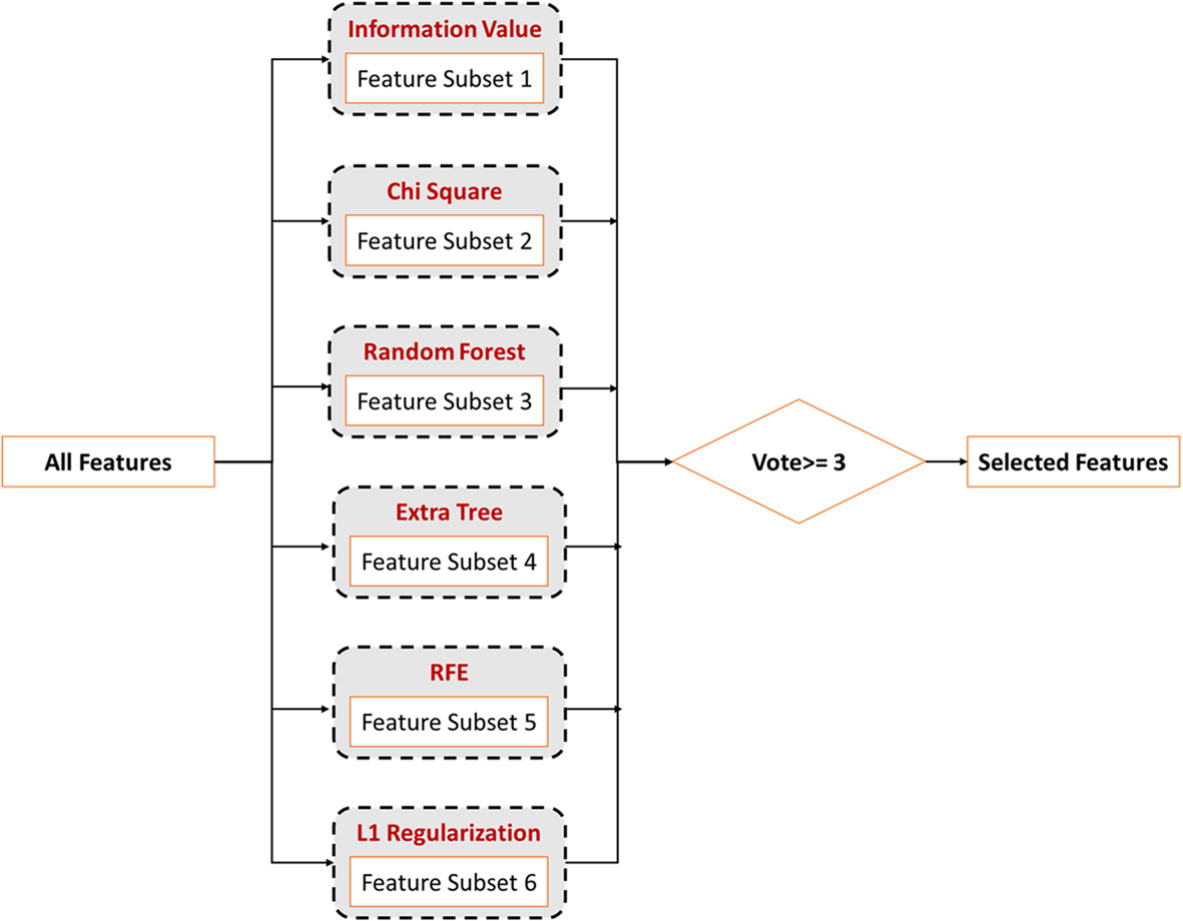
Integration of multiple feature selection techniques via voting approach

For each of the preceding feature selection methods, we sort the features by their absolute values, retaining the top half while discarding the remainder. Then, we identify the features chosen by three or more of these techniques, as shown in Algorithm 2. This voting approach, which combines multiple feature selectors to obtain a final feature subset, is depicted in Fig. 4. These selected features are then continued to subsequent stages of the methodology.

**Table:**
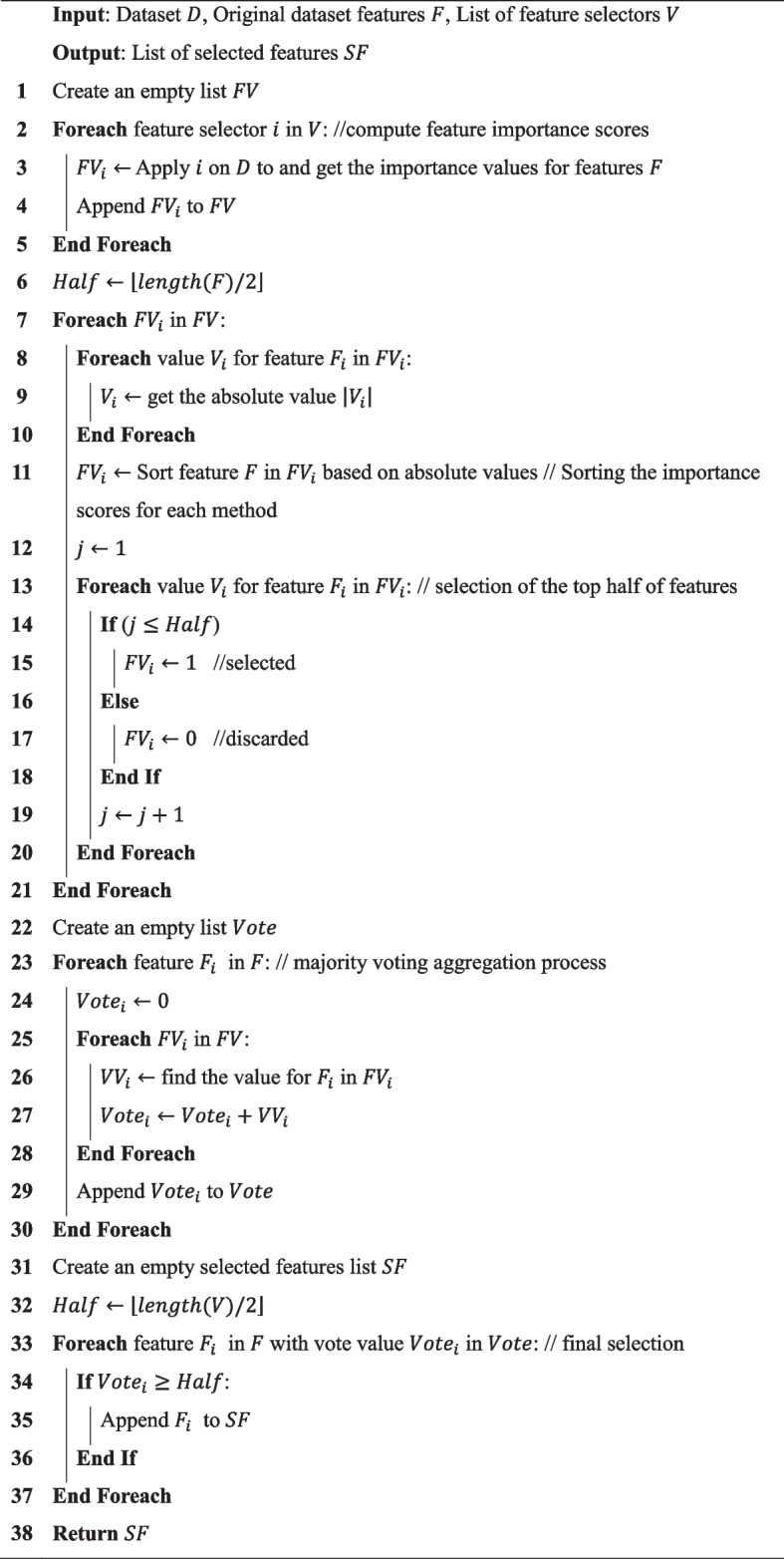
Algorithm 2: Feature selection via majority voting

The computational complexity of the proposed feature selection method can be analyzed by examining its key operations. Let $N$ denote the number of features in the dataset and $M$ represent the number of feature selection methods applied. The algorithm first applies each feature selection method to compute feature importance scores, which requires $\text{\rm O}(N.M)$ operations. Sorting the importance scores for each method has a complexity of $\text{\rm O}(N\text{ log}N)$, leading to an overall complexity of this step $\text{\rm O}(M.N\text{ log}N)$. The subsequent selection of the top half of features is a linear operation, contributing $\text{\rm O}(M.N)$. The majority voting process iterates over all features and feature selection methods, requiring $\text{\rm O}(N.M)$ for aggregation and another $\text{\rm O}(N)$ for final selection. Thus, the dominant term in the complexity analysis arises from the sorting step, resulting in an overall computational complexity of $\text{\rm O}(M.N\text{ log}N)$. This ensures that the feature selection process remains efficient, even for large-scale datasets, while leveraging ensemble-based selection to improve robustness and stability.

### Model training

The reduced datasets are split into 90% for model training and 10% for testing, and the training datasets are utilized to establish seven distinct models, each tailored to one of the seven datasets. These models include XGBoost, RF, ET, and QDA for classification. XGBoost [37] is a refined distributed gradient boosting framework that defines a loss function and systematically generates new trees, prioritizing minimizing this loss function to improve predictive accuracy. The formulation of XGBoost can be expressed as shown in Eq. (7).

7 $${\widehat{y}}_{i}=\sum_{k=1}^{n}{f}_{k}({x}_{i}), {f}_{k}\in F$$

where $F$ denotes the space of trees, ${f}_{k}({x}_{i})$ is the result of tree $k$, and ${\widehat{y}}_{i}$ is the predicted value of $i$-th instance ${x}_{i}$. The goal is to minimize the loss function, as Eq. (8) indicates.

8 $$obj\left(\theta \right)=\sum_{i}l\left({\widehat{y}}_{i},{y}_{i}\right)+ \sum_{k}\Omega \left({f}_{k}\right)$$

where $l$ is the loss function, $\Omega$ represents a penalty value that aims to balance model complexity and predictive accuracy. By adjusting the value of $\Omega$, the algorithm can control the level of regularization applied to the model, which reduces overfitting and improves generalization to unseen data. A higher $\Omega$ value encourages a simpler model with fewer leaves. A lower $\Omega$ value allows for a more complex model with more leaves, ultimately impacting the model's overall performance.

In RF [33], $k$ subsets are randomly drawn from the training dataset using the bootstrap technique. Each of these $k$ subsets is utilized to construct a distinct classification decision tree. This is achieved by iteratively selecting a random subset of features, and the optimal feature within this subset is determined by calculating information gain. The selected feature guides the node to be split into two child nodes, and this recursive process continues until the tree reaches its maximum allowable size. This procedure is repeated $k$ times, resulting in the generation of $k$ trees. These trees collectively form the RF ensemble, and final predictions are obtained by aggregating the outputs of multiple tree classifiers using a majority voting approach. In a binary classification problem, the majority voting method can be expressed by Eq. (9).

9 $$\widehat{y}=\left\{\begin{array}{c}1, {\sum }_{i=0}^{k}{y}_{i}\ge \theta ,\\ 0, {\sum }_{i=0}^{k}{y}_{i}<\theta \end{array}\right.$$

where $\widehat{y}$ is the final result, ${y}_{i}$ is $i$-th tree’s result, and $\theta$ is a threshold.

The process of ET [38] shares similarities with RF but exhibits distinct characteristics. Both methods involve drawing $k$ subsets randomly from the training set using bootstrapping. However, in ET, the process extends to selecting random split thresholds for features, unlike RF, which relies solely on optimal thresholds. Despite this difference, the creation process of decision trees in ET mirrors that of RF, resulting in the formation of the ET ensemble. However, there is a notable distinction in how predictions are combined: while RF employs majority voting, ET combines predictions across trees with equal weight, thus enhancing the ensemble's predictive diversity.

QDA [39] operates by modeling the probability distributions of different classes based on the features of datasets. QDA allows each class to have its covariance matrix and creates quadratic decision boundaries between classes. Assume a simple max gate function $g\left(X\right)$ as a classification rule. The prior probability of class $i$ is ${p}_{i}$, and the conditional density of feature $X$ in class $i$ is ${f}_{i}\left(X\right)$. It is assumed that the feature vector $X$ is multivariate normally distributed in the group with mean ${\mu }_{i}$ and class covariance matrix ${M}_{i}$. It is also assumed that ${g}_{i}\left(X\right)>{g}_{j}\left(X\right)$ for $j\ne i$. Then, ${f}_{i}\left(X\right)$ can be calculated by Eq. (10). Also, the maximum a-posteriori discriminant function can be calculated as in Eq. (11).

10 $${f}_{i}\left(X\right)=\frac{1}{2p{\left|{M}_{i}\right|}^\frac{1}{2}}exp\left[-\frac{1}{2}{\left(X-{\mu }_{i}\right)}^{T}\sum_{i}^{-1}\left(X-{\mu }_{i}\right)\right]$$

11 $${g}_{i}\left(X\right)=-\frac{1}{2}{\left(X-{\mu }_{i}\right)}^{T}\sum_{i}^{-1}\left(X-{\mu }_{i}\right)-\frac{1}{2}\text{log}\left(\left|{M}_{i}\right|\right)+\text{log}\left({p}_{i}\right)$$

A voting ensemble classifier [40] is a popular technique in machine learning that combines the predictions of multiple individual classifiers to make a final prediction. There are two main types of voting ensembles: hard voting and soft voting. In hard voting, each classifier casts a single vote for its predicted class, and the class with the most votes is chosen as the final prediction. In soft voting, each classifier assigns a probability score to each class, and the average probabilities across all classifiers are used to make the final prediction. Soft voting is utilized in the proposed methodology. Algorithm 3 shows the soft voting procedure employed for binary classification across the proposed multilevel cancer classification system.

**Fig. 5:**
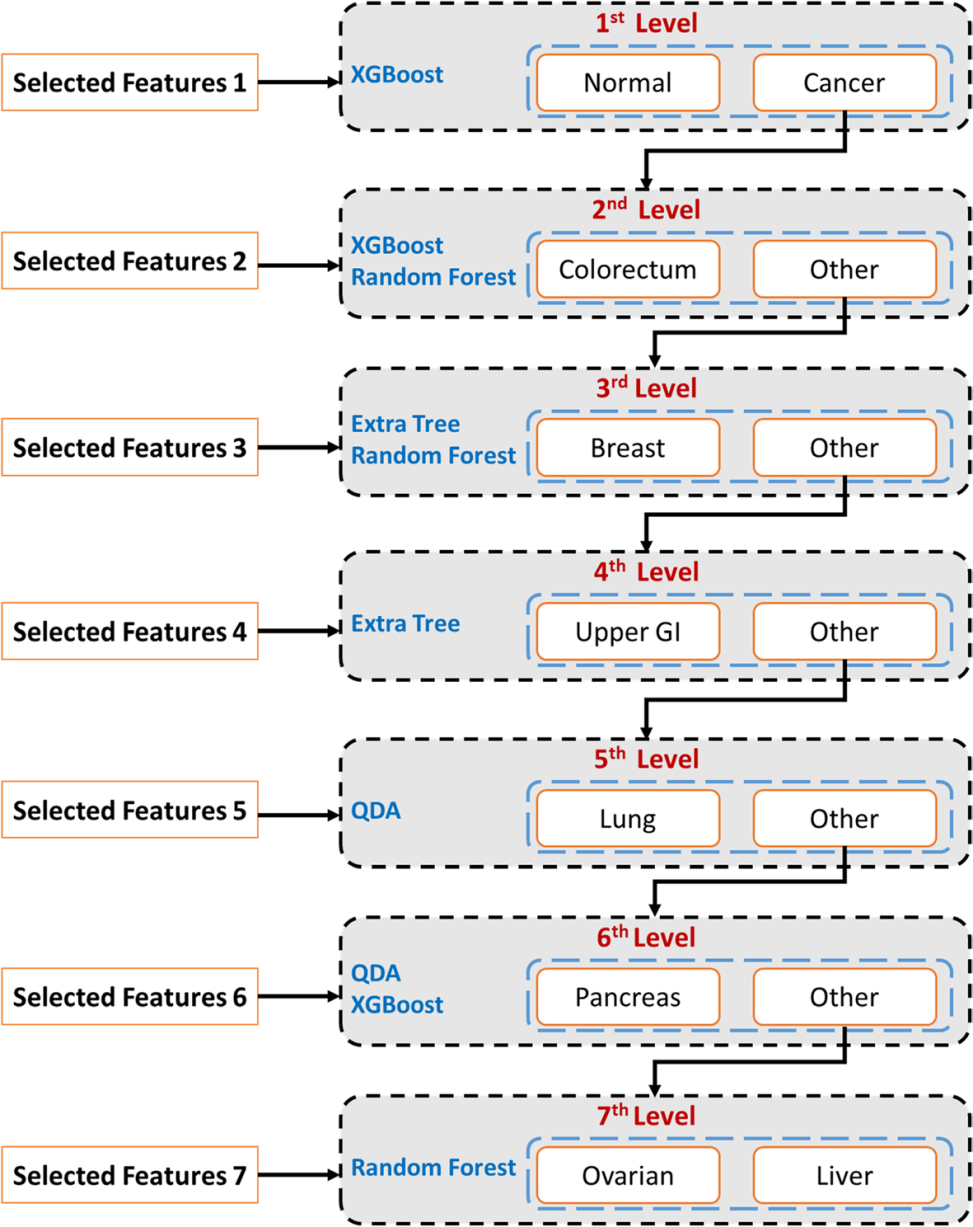
Procedure for the proposed multi-level cancer classification system

Figure 5 shows the multi-level cancer classification process employed in the proposed methodology, structured to differentiate between various cancer types effectively. At the initial level, XGBoost is utilized to discern between individuals with normal conditions and those with cancer. Subsequently, a soft voting ensemble comprising XGBoost and RF is deployed at the second level to classify patients with colorectal cancer from other patient groups. Moving to the third level, a soft voting ensemble of ET and RF is employed to classify breast cancer patients accurately. At the fourth level, ET is exclusively used to classify patients with upper GI cancer. Transitioning to the fifth level, QDA categorizes lung cancer patients.

**Table:**
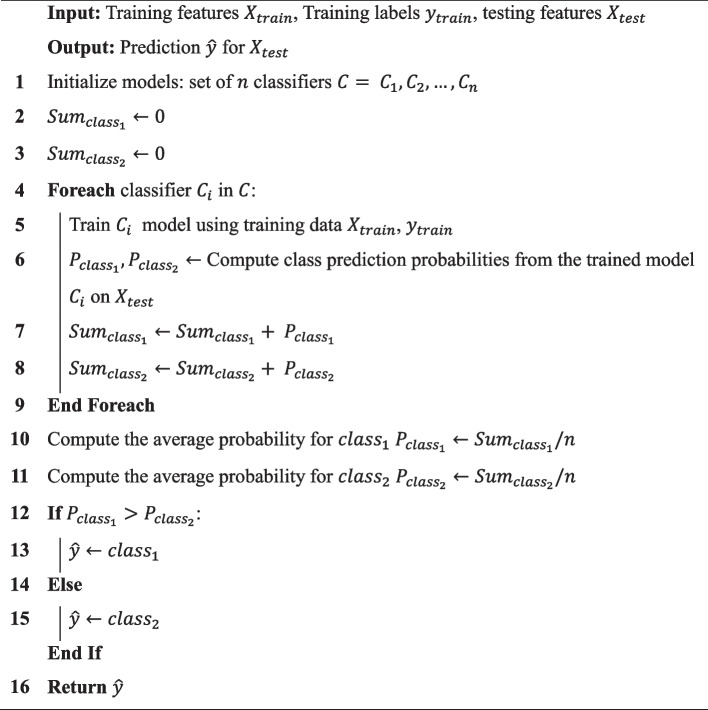
Algorithm 3: Ensemble soft voting for binary classification

At the sixth level, a soft voting ensemble of QDA and XGBoost is utilized to classify patients with pancreatic cancer. Finally, at the seventh level, RF is used to differentiate ovarian and liver cancer patients. This systematic approach, integrating various classifiers at different levels, enhances the accuracy and robustness of the cancer classification process, contributing to improved patient care and treatment strategies.

### Model evaluation

The model's performance is evaluated based on its predictive results, where True Positives (TP) represent correctly classified instances within the positive class, and False Positives (FP) refer to instances incorrectly classified within the positive class. Conversely, True Negatives (TN) indicate correctly classified instances within the negative class, while False Negatives (FN) denote instances incorrectly classified within the negative class.

Equation (12) calculates model accuracy [41] by dividing the number of true classifications by the total number of classifications. Precision measures the proportion of correctly identified positive samples among all predicted positives (Eq. 13), where lower precision suggests a higher occurrence of false positives. Recall, also referred to as the True Positive Rate (TPR) or sensitivity, evaluates the proportion of actual positive samples that are correctly identified (Eq. 14), with higher recall indicating fewer misclassified positive cases. The F1-score, derived from Eq. 15, represents the harmonic mean of precision and recall, providing a balanced assessment of the model’s performance [42].

12 $$Accuracy (Acc)=\frac{TP+TN}{TP + FP+ FN +TN}$$

13 $$Precision=\frac{TP}{TP+FP}$$

14 $$Recall=\frac{TP}{TP+FN}$$

15 $$F1-score (F1)=\frac{2*Precision*Recall}{Precision+Recall}$$

True Negative Rate (TNR), known as specificity, quantifies the proportion of correctly classified negative samples among all negatives (Eq. 16). Balanced accuracy is computed as the average of the TPR and the TNR. This metric is especially valuable when dealing with imbalanced datasets, as it provides a more reliable assessment of model performance. Balanced accuracy can be determined using Eq. 17 [43].

16 $$Specificity=\frac{TN}{TN+FP}$$

17 $$Balanced Accuracy (BA)=\frac{Recall+Specificity}{2}$$

The current study also uses the AUC [41], the area under the Receiver Operating Characteristic (ROC) curve. The ROC curve is produced by adjusting the x-axis False Positive Rate (FPR) and y-axis TPR. FPR is computed as the ratio of incorrectly predicted negative instances employing Eq. 18. AUC values vary from 0 to 1, with 1 indicating that the model can adequately discriminate between negative and positive classes 100% of the time.

18 $$FPR=\frac{FP}{FP + TN}$$

## Experimental results

The experimental results were obtained using a PC equipped with an Intel i5-8250U CPU, a 2GB Nvidia GeForce MX130 GPU, and 12GB of RAM, running Windows 10. The proposed system and experiments were implemented in Python 3.9.6, utilizing the scikit-learn 0.22, CatBoost 1.2, LightGBM 3.3.2, MEALPY 3.0.1, and SHAP 0.42.1 libraries. The proposed approach makes use of 43 features in total, including 39 cfDNA/ctDNA biomarker features, three clinical characteristics (ethonic, age, and sex), and an omega score feature, which are utilized in different phases of the methodology, and the results from each phase are passed on to the subsequent phase to produce the final classification results.

**Fig. 6:**
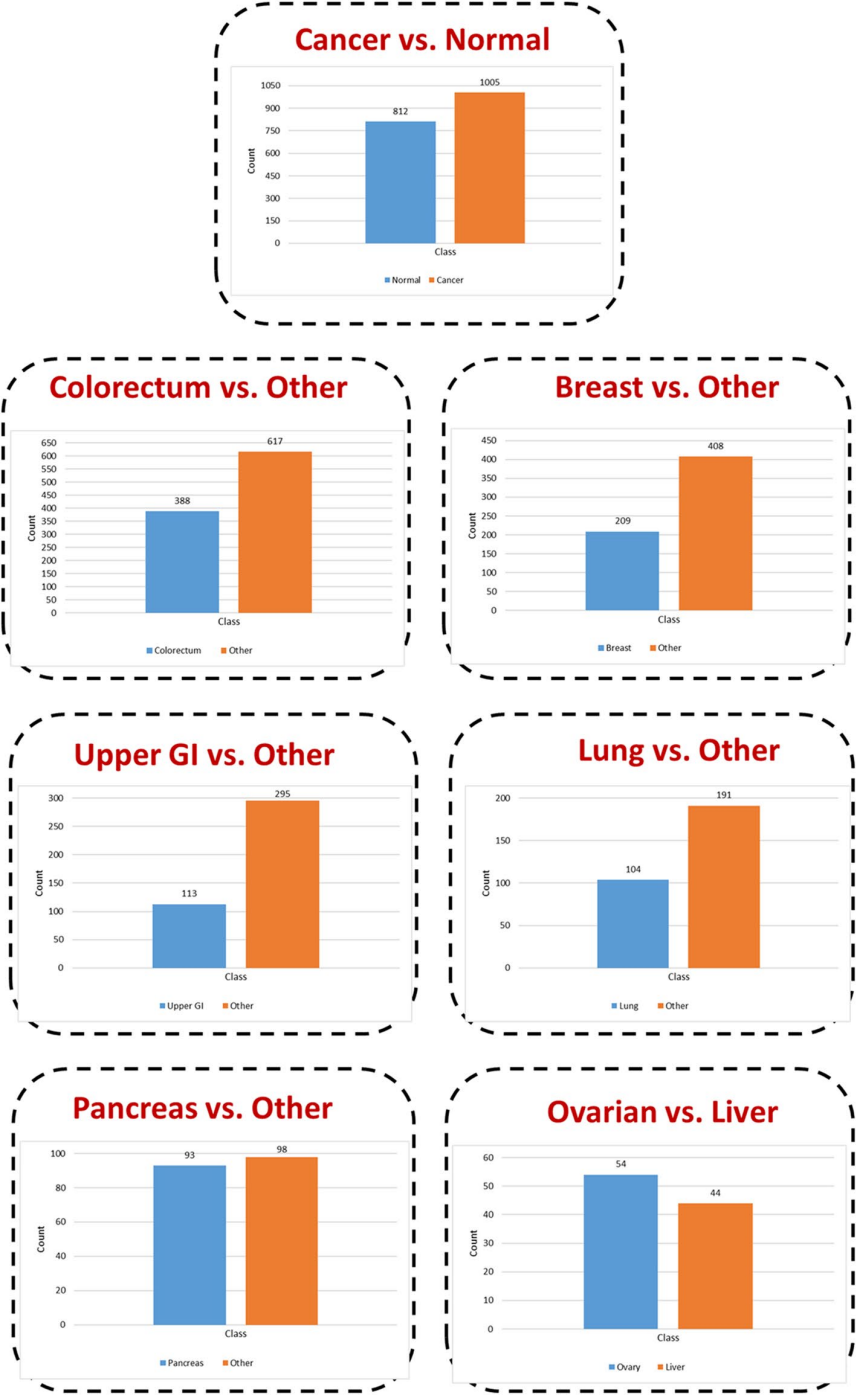
Class distribution across seven distinct datasets

Initially, we generated seven distinct datasets, each aligned with a specific classification level. Figure 6 illustrates the class distribution across these datasets. This approach involves decomposing a multi-class classification problem into multiple binary classification tasks, simplifying the complexity by transforming it into a sequence of binary decisions to distinguish between two classes. Moreover, isolating each class from the others enables a more concentrated examination of class-specific features and decision boundaries, ultimately enhancing model performance and providing deeper insights. Table 3 presents the training and testing data distribution after performing the train-test split.

**Table 3:** Distribution of training and testing data across classification tasks

Dataset	Training Data	Testing Data	Total Count	Overall
Normal vs. Cancer	731	81	812	1817
	904	101	1005	
Colorectum vs. Other	349	39	388	1005
	555	62	617	
Breast vs. Other	188	21	209	617
	367	41	408	
Upper GI vs. Other	102	11	113	408
	265	30	295	
Lung vs. Other	93	11	104	295
	172	19	191	
Pancreas vs. Other	83	10	93	191
	88	10	98	
Ovarian vs. Liver	48	6	54	98
	40	4	44	

### Feature selection results

Feature selection is implemented on the seven datasets using a voting mechanism among six feature selection methods. This approach identifies and retains the most informative features while discarding less important ones. The feature selection process facilitates a comprehensive exploration of the feature space, capturing diverse aspects of feature relevance. Each technique contributes unique strengths to the selection process, considering various statistical and algorithmic factors. Prioritizing features with strong discriminatory power, the strategy retains the highest half of features based on absolute feature values.

As stated before, IV is based on the WoE method, in which we used monotonic binning. Table 4 presents the key hyperparameters used in the WOE transformation, detailing their respective functions and values. The max_bins parameter restricts the maximum number of bins to 20, balancing granularity and model complexity. These hyperparameters contribute to a structured and robust feature encoding process, which is essential for improving predictive performance in machine learning models.

**Table 4:** Hyperparameters of the IV method

hyperparameter	Description	Value
cardinality	Minimum number of unique values required for monotonic binning	5
bins	Number of bins enforced for monotonic binning	3
max_bins	Maximum number of bins allowed for monotonic binning	20

We utilized the default hyperparameter settings provided by scikit-learn for the Chi-Square feature selection method. For feature selection using the random forest method, Table 5 presents the key hyperparameters of the technique, detailing their functions and values. With n_estimators = 100, meaning the model consists of 100 decision trees. The feature importance scores were derived using the Gini impurity, which measures the discriminatory power of each feature. The model employed bootstrap sampling, enhancing generalization by training each tree on a randomly sampled subset of the dataset. These settings collectively improve the reliability of the selected features for downstream tasks.

**Table 5:** Hyperparameters of the random forest feature selection method

hyperparameter	Description	Value
n_estimators	Number of decision trees in the forest	100
criterion	Function to measure split quality	Gini
min_samples_split	Minimum number of samples to split an internal node	2
min_samples_leaf	Minimum number of samples required at a leaf node	1
bootstrap	Whether bootstrap sampling is used when building trees	True

We utilized the same hyperparameters of the random forest methods for feature selection using the extra tree method, with two key differences. First, extra tree does not use bootstrap sampling (bootstrap = False), meaning each tree is trained on the full dataset rather than a random subset. Second, instead of searching for the best split, the extra tree selects split points at random, leading to greater variance reduction. These settings make extra trees a powerful and efficient technique for feature selection.

For RFE feature selection, we used Logistic Regression as the base estimator. RFE iteratively removes the least important features, retraining the model at each step until the desired number of features (21) is selected. The base estimator is configured with L2 regularization and the 'lbfgs' solver. The L2 regularization in Logistic Regression helps prevent overfitting, while the lbfgs solver ensures efficient optimization. Table 6 presents the hyperparameters used for RFE and its Logistic Regression estimator.

**Table 6:** Hyperparameters of RFE with logistic regression

hyperparameter	Description	Value
step	Number of features removed at each iteration	1
penalty	Regularization type	L2
C	Inverse regularization strength	1.0
solver	Optimization algorithm	lbfgs
max_iter	Maximum number of optimization iterations	100
tolerance	Stopping criterion for convergence	0.0001

For L1 regularization feature selection, we used linear SVM as the base model. The L1 regularization helps in sparsity promotion, encouraging the model to set some feature weights to exactly zero, effectively eliminating irrelevant features. This makes it a useful approach for selecting the most important features (21) while improving interpretability and reducing overfitting. The hyperparameters used in our implementation are summarized in Table 7. The choice of C = 0.01 ensures stronger regularization, leading to a more compact feature set while preventing overfitting. This approach effectively reduces dataset dimensionality while preserving key features contributing to accurate classification.

**Table 7:** Hyperparameters of L1-regularized linear SVC for feature selection

hyperparameter	Description	Value
C	Inverse regularization strength	0.01
loss	The loss function used for optimization	squared_hinge
max_iter	Maximum number of optimization iterations	1000
tolerance	Stopping criterion for convergence	0.0001

We systematically recorded the feature importance values across the six techniques for each dataset and voting method. These importance values were compiled to assess the contribution of each biomarker to the classification task. Then, the voting mechanism was implemented, where each feature received a vote based on its selection by the different methods. The total number of votes determined the overall significance of a feature, facilitating the identification of the most relevant biomarkers. The final results, detailing the importance of values and voting outcomes, were compiled and made available as a supplementary resource (Online Resource 2).

A threshold of three or higher votes for feature inclusion ensures the conservation of features with significant influence across multiple methods, reducing bias inherent in single-feature selection techniques. This approach enhances the robustness of feature selection by promoting consensus among diverse methodologies. The selected features for each dataset are summarized in Table 8.

**Table 8:** Selected features at each level of the multi-level cancer classification system

Dataset	Selected Features
Normal vs. Cancer	IL-8, IL-6, HGF, sEGFR, Age, TGFa, ethonic, Omega Score, Prolactin, Myeloperoxidase, FGF2, CA19-9, CA-125, OPN, HE4, TIMP-1, NSE, CYFRA 21–1, GDF15, DKK1, CEA, sFas, OPG, G-CSF
Colorectum vs. Other	CA 15–3, CA-125, TGFa, sFas, SHBG, OPG, CA19-9, CD44, Age, NSE, Kallikrein-6, Follistatin, IL-6, HGF, G-CSF, Leptin, AXL, AFP, IL-8, TIMP-2, Thrombospondin-2, CYFRA 21–1, gender, sHER2/sEGFR2/sErbB2
Breast vs. Other	IL-6, Age, gender, CA-125, NSE, GDF15, HE4, Mesothelin, Omega Score, PAR, HGF, CA 15–3, CA19-9, Thrombospondin-2, OPN, TIMP-1, OPG, Leptin, Midkine, sHER2/sEGFR2/sErbB2, IL-8, Myeloperoxidase, Galectin-3
Upper GI vs. Other	sHER2/sEGFR2/sErbB2, G-CSF, HGF, Galectin-3, Myeloperoxidase, Thrombospondin-2, CD44, TGFa, sEGFR, IL-8, Kallikrein-6, CA 15–3, Mesothelin, ethonic, Prolactin, Midkine, TIMP-2, NSE, IL-6, SHBG, CA-125, CA19-9, gender, Leptin, OPN
Lung vs. Other	NSE, HGF, CA19-9, sHER2/sEGFR2/sErbB2, Myeloperoxidase, CA-125, HE4, sFas, AXL, G-CSF, TIMP-1, IL-8, FGF2, IL-6, OPN, TIMP-2, GDF15, AFP, TGFa, OPG, SHBG, Galectin-3
Pancreas vs. Other	sHER2/sEGFR2/sErbB2, Myeloperoxidase, CA-125, G-CSF, CD44, Omega Score, AXL, Midkine, CA19-9, Galectin-3, TIMP-2, sPECAM-1, sEGFR, AFP, Angiopoietin-2, CEA, Prolactin, FGF2, IL-6, Mesothelin, sFas, NSE, Thrombospondin-2, ethonic
Ovarian vs. Liver	CA-125, HE4, AFP, OPN, Thrombospondin-2, sFas, G-CSF, Endoglin, SHBG, Kallikrein-6, sEGFR, HGF, TIMP-2, TGFa, Follistatin, gender, IL-8, Myeloperoxidase, ethonic, Mesothelin, PAR, sPECAM-1, CEA, Prolactin

The proposed method identified 24, 24, 23, 25, 22, 24, and 24 features for the respective seven datasets. The training and testing datasets containing the selected features for all seven levels are provided as pickle files. These files ensure efficient storage and retrieval while preserving data structures. They are available in supplementary information (Online Resource 3), allowing for reproducibility and further analysis. These selected features play a crucial role in enhancing the interpretability of the proposed system. By highlighting these key features, clinicians can gain valuable insights into the underlying patterns influencing cancer classification, which improves the clinical relevance and utility of the developed models, aiding in more informed decision-making processes.

### Model training results

The selected features from each dataset are then fed into the subsequent stage of the proposed multi-level cancer classification system. Utilizing XGBoost, RF, ET, and QDA, individually or in ensemble configurations through soft voting, forms the core of our approach. For each of the seven levels in the proposed system, the designated model was trained using tenfold cross-validation (CV) on the training set. The model that achieved the best performance in cross-validation was subsequently used for evaluation on the testing set.

At the first level, XGBoost was employed as the classification model. The results of the tenfold CV for XGBoost are presented in Table 9, with an average accuracy of 98.35% and an average AUC of 99.74%. The hyperparameters of the best-performing model, as determined by cross-validation, are summarized in Table 10. The model uses "gbtree" booster, meaning the model uses a tree-based boosting approach, and the number of boosting rounds to optimize predictive performance is 100.

**Table 9:** Performance metrics from 10-Fold CV for the first level

Fold	Acc	Precision	Recall	F1	Specificity	BA	AUC
1	0.98171	0.98889	0.97802	0.98343	0.98630	0.98216	0.99277
2	0.98780	1.00000	0.97802	0.98889	1.00000	0.98901	0.99970
3	0.97561	0.95789	1.00000	0.97849	0.94521	0.97260	0.99804
4	0.98780	0.97849	1.00000	0.98913	0.97260	0.98630	0.99819
5	1.00000	1.00000	1.00000	1.00000	1.00000	1.00000	1.00000
6	0.98773	0.98889	0.98889	0.98889	0.98630	0.98760	0.99376
7	0.98773	0.97826	1.00000	0.98901	0.97260	0.98630	0.99848
8	0.96319	0.96667	0.96667	0.96667	0.95890	0.96279	0.99741
9	0.97546	1.00000	0.95556	0.97727	1.00000	0.97778	0.99772
10	0.98773	0.98889	0.98889	0.98889	0.98630	0.98760	0.99756
**Mean**	0.98348	0.98480	0.98560	0.98507	0.98082	0.98321	0.99736
**Std**	0.01003	0.01445	0.01573	0.00909	0.01849	0.01018	0.00233

**Table 10:** XGBoost hyperparameters for the first level

hyperparameter	Description	Value
booster	Specifies the boosting algorithm to use	gbtree
base_score	Initial prediction score before boosting starts	0.5
learning_rate	Step size shrinkage to prevent overfitting	0.3
max_depth	Maximum depth of a tree	6
min_child_weight	The minimum sum of instance weight needed in a child	1
n_estimators	Number of boosting rounds (trees)	100

The second level of the proposed system utilizes a soft voting ensemble combining XGBoost and Random Forest classifiers. This ensemble approach leverages the strengths of both models, where predictions are aggregated based on the probability scores assigned by each classifier. The tenfold CV results obtained during training are presented in Table 11, with an average accuracy of 83.52% and an average AUC of 92.11%. The hyperparameters for XGBoost remain consistent with those listed in Table 10, while the hyperparameters for Random Forest are consistent with those previously stated in Table 5.

**Table 11:** Performance metrics from 10-Fold CV for the second level

Fold	Acc	Precision	Recall	F1	Specificity	BA	AUC
1	0.84615	0.85000	0.91071	0.87931	0.74286	0.82679	0.89592
2	0.80220	0.78788	0.92857	0.85246	0.60000	0.76429	0.91071
3	0.84615	0.86207	0.89286	0.87719	0.77143	0.83214	0.93265
4	0.84615	0.90385	0.83929	0.87037	0.85714	0.84821	0.93776
5	0.83333	0.82258	0.92727	0.87179	0.68571	0.80649	0.94961
6	0.82222	0.80952	0.92727	0.86441	0.65714	0.79221	0.90442
7	0.81111	0.80645	0.90909	0.85470	0.65714	0.78312	0.85922
8	0.78889	0.80000	0.87273	0.83478	0.65714	0.76494	0.91377
9	0.91111	0.94340	0.90909	0.92593	0.91429	0.91169	0.96260
10	0.84444	0.81818	0.96429	0.88525	0.64706	0.80567	0.94433
**Mean**	0.83518	0.84039	0.90812	0.87162	0.71899	0.81355	0.92110
**Std**	0.03366	0.05000	0.03422	0.02424	0.10136	0.04427	0.03053

The third level of the proposed system employs a soft voting ensemble that combines Extra Trees and Random Forest classifiers. This ensemble strategy enhances model robustness by incorporating diverse tree-based learners, reducing variance, and improving generalization. The tenfold CV results for this level are presented in Table 12, with an average accuracy of 87.22% and an average AUC of 94.63%. The hyperparameters for Extra Trees and Random Forest remain consistent with those stated during the feature selection phase.

**Table 12:** Performance metrics from 10-Fold CV for the third level

Fold	Acc	Precision	Recall	F1	Specificity	BA	AUC
1	0.875	0.89474	0.91892	0.90667	0.78947	0.85420	0.92532
2	0.875	0.875	0.94595	0.90909	0.73684	0.84139	0.94808
3	0.80357	0.88235	0.81081	0.84507	0.78947	0.80014	0.91465
4	0.83929	0.86842	0.89189	0.88	0.73684	0.81437	0.92248
5	0.875	0.89474	0.91892	0.90667	0.78947	0.85420	0.95804
6	0.89091	0.91892	0.91892	0.91892	0.83333	0.87613	0.97147
7	0.92727	0.92308	0.97297	0.94737	0.83333	0.90315	0.97973
8	0.87273	0.93939	0.86111	0.89855	0.89474	0.87792	0.92398
9	0.90909	0.91892	0.94444	0.93151	0.84211	0.89327	0.96857
10	0.85455	0.86842	0.91667	0.89189	0.73684	0.82675	0.95102
**Mean**	0.87224	0.89840	0.91006	0.90357	0.79825	0.85415	0.94633
**Std**	0.03476	0.0253	0.04629	0.02813	0.05296	0.03398	0.02338

The fourth level of the proposed system employs Extra Trees as the sole classification model. This decision tree-based ensemble method is known for efficiently handling high-dimensional data while reducing overfitting through randomized feature selection and splitting. The tenfold CV results for this level are summarized in Table 13, with an average accuracy of 85.82% and an average AUC of 92.06%. The hyperparameters for Extra Trees remain consistent with those stated during the feature selection phase.

**Table 13:** Performance metrics from 10-Fold CV for the fourth level

Fold	Acc	Precision	Recall	F1	Specificity	BA	AUC
1	0.97297	1	0.9	0.94737	1	0.95	0.96481
2	0.83784	1	0.4	0.57143	1	0.7	0.84444
3	0.70270	0.44444	0.4	0.42105	0.81481	0.60741	0.82037
4	0.94595	1	0.8	0.88889	1	0.9	0.96296
5	0.83784	0.75	0.6	0.66667	0.92593	0.76296	0.94074
6	0.89189	0.88889	0.72727	0.8	0.96154	0.84441	0.96678
7	0.86486	0.8	0.72727	0.76190	0.92308	0.82517	0.95979
8	0.86111	0.72727	0.8	0.76190	0.88462	0.84231	0.93077
9	0.86111	0.77778	0.7	0.73684	0.92308	0.81154	0.89423
10	0.80556	0.8	0.4	0.53333	0.96154	0.68077	0.92115
**Mean**	0.85818	0.81884	0.64545	0.70894	0.93946	0.79246	0.92061
**Std**	0.07442	0.16966	0.18611	0.16273	0.05891	0.10495	0.05214

The fifth level of the proposed system utilizes QDA as the classification model. QDA is a probabilistic model that assumes different covariance structures for each class, making it suitable for datasets where class distributions exhibit varying variances. The tenfold CV results for this level are reported in Table 14, with an average accuracy of 85.29% and an average AUC of 91.86%. The hyperparameters used for QDA a regularization parameter (reg_param) set to 0.0, ensuring no shrinkage is applied to the covariance estimates. Additionally, class priors are inferred from the data, allowing the model to adapt to class distributions automatically. The tolerance (tol) is set to 0.0001, defining the threshold for rank estimation during computation. These settings enable QDA to capture class-specific covariance structures.

**Table 14:** Performance metrics from 10-Fold CV for the fifth level

Fold	Acc	Precision	Recall	F1	Specificity	BA	AUC
1	0.81481	0.93333	0.77778	0.84848	0.88889	0.83333	0.90741
2	0.92593	0.94444	0.94444	0.94444	0.88889	0.91667	0.91667
3	0.88889	0.9375	0.88235	0.90909	0.9	0.89118	0.88235
4	0.92593	0.89474	1	0.94444	0.8	0.9	0.95294
5	0.70370	0.84615	0.64706	0.73333	0.8	0.72353	0.76471
6	0.80769	0.92857	0.76471	0.83871	0.88889	0.82680	0.96732
7	0.80769	0.83333	0.88235	0.85714	0.66667	0.77451	0.89869
8	0.92308	1	0.88235	0.9375	1	0.94118	1
9	0.80769	0.92857	0.76471	0.83871	0.88889	0.82680	0.97386
10	0.92308	0.94118	0.94118	0.94118	0.88889	0.91503	0.92157
**Mean**	0.85285	0.91878	0.84869	0.87930	0.86111	0.85490	0.91855
**Std**	0.07576	0.04906	0.10740	0.06883	0.08815	0.06991	0.06563

The sixth level of the proposed system employs a soft voting ensemble that combines Quadratic Discriminant Analysis (QDA) and XGBoost to enhance classification performance. This ensemble leverages the strengths of both models, balancing XGBoost's robust feature handling with QDA's probabilistic decision boundaries. The tenfold CV training results for this level are presented in Table 15, with an average accuracy of 86.11 and an average AUC of 96.91%. The hyperparameters for both models remain consistent with those stated in the previous levels.

**Table 15:** Performance metrics from 10-Fold CV for the sixth level

Fold	Acc	Precision	Recall	F1	Specificity	BA	AUC
1	0.61111	0.625	0.55556	0.58824	0.66667	0.61111	0.80247
2	0.88235	0.8	1	0.88889	0.77778	0.88889	1
3	0.82353	0.72727	1	0.84211	0.66667	0.83333	1
4	0.94118	0.88889	1	0.94118	0.88889	0.94444	0.98611
5	0.88235	0.8	1	0.88889	0.77778	0.88889	0.97222
6	0.76471	0.83333	0.625	0.71429	0.88889	0.75694	0.95833
7	0.94118	1	0.875	0.93333	1	0.9375	0.98611
8	0.94118	0.88889	1	0.94118	0.88889	0.94444	1
9	0.94118	0.9	1	0.94737	0.875	0.9375	1
10	0.88235	0.88889	0.88889	0.88889	0.875	0.88194	0.98611
**Mean**	0.86111	0.83523	0.89444	0.85743	0.83056	0.8625	0.96914
**Std**	0.10552	0.10452	0.16823	0.11740	0.10654	0.10627	0.06017

The seventh and final level of the proposed system employs Random Forest as a standalone model for classification. RF is leveraged for its ensemble learning capability, which enhances predictive accuracy and reduces overfitting by aggregating multiple decision trees. The tenfold CV training results for this level are presented in Table 16, with an average accuracy of 97.78% and an average AUC of 1. The hyperparameters for RF remain consistent with those previously stated.

**Table 16:** Performance metrics from 10-Fold CV for the seventh level

Fold	Acc	Precision	Recall	F1	Specificity	BA	AUC
1	1	1	1	1	1	1	1
2	0.88889	1	0.8	0.88889	1	0.9	1
3	1	1	1	1	1	1	1
4	1	1	1	1	1	1	1
5	1	1	1	1	1	1	1
6	1	1	1	1	1	1	1
7	1	1	1	1	1	1	1
8	0.88889	1	0.8	0.88889	1	0.9	1
9	1	1	1	1	1	1	1
10	1	1	1	1	1	1	1
**Mean**	0.97778	1	0.96	0.97778	1	0.98	1
**Std**	0.04685	0	0.08433	0.04685	0	0.04216	0

The average training results across the seven levels are 89.16%, 89.95%, 87.89%, 88.34%, 87.56%, 87.73%, and 95.33% for accuracy, precision, recall, F1-score, specificity, balanced accuracy, and AUC respectively. The trained models used for evaluation are stored as pickle files to preserve their structure and parameters. These files facilitate easy deployment and reproducibility of the results. They are available in supplementary information (Online Resource 4), allowing researchers to analyze further and validate the models.

### Model evaluation results

**Fig. 7:**
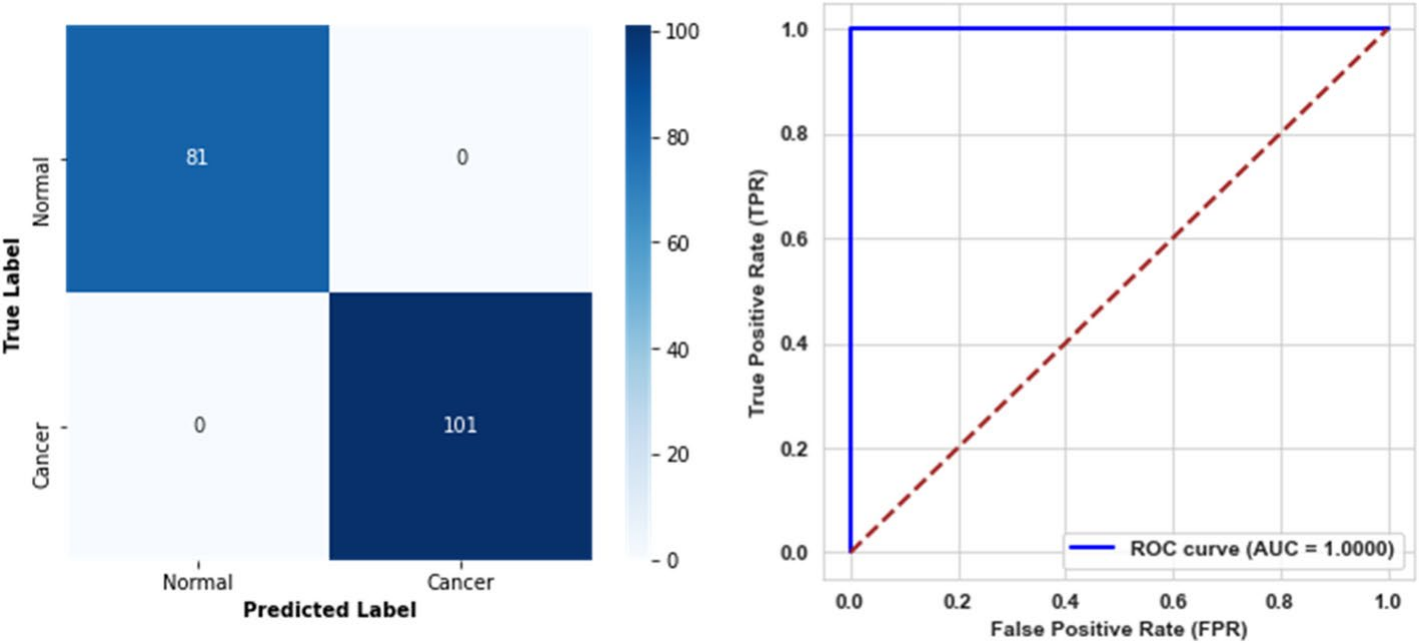
Confusion matrix and ROC curve for the first level model

The best-performing model from the training phase was selected for the evaluation step on the designated testing sets. The evaluation results for the first level demonstrate performance across all metrics, achieving performance across all evaluation metrics, with all scores reaching 100%. The confusion matrix and the ROC curve, illustrated in Fig. 7, further confirm the model's effectiveness in distinguishing between classes.

**Fig. 8:**
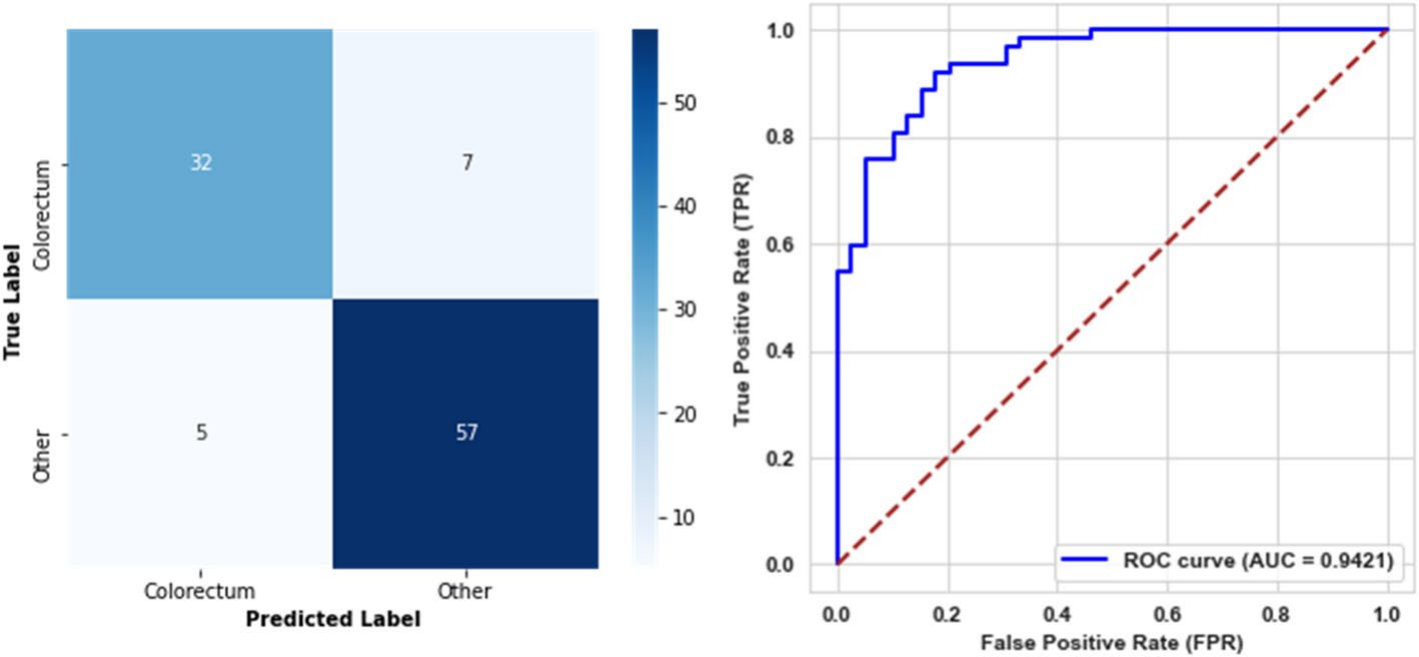
Confusion matrix and ROC curve for the second level model

For the second level, the trained model was evaluated on the testing set and achieved an accuracy of 88.12%, a precision of 89.06%, a recall of 91.94%, and an F1-score of 90.48%. Additionally, it demonstrated a specificity of 82.05%, a balanced accuracy of 86.99%, and an AUC of 94.21%. These results indicate a promising predictive performance. The confusion matrix and the AUC curve for this model are presented in Fig. 8.

**Fig. 9:**
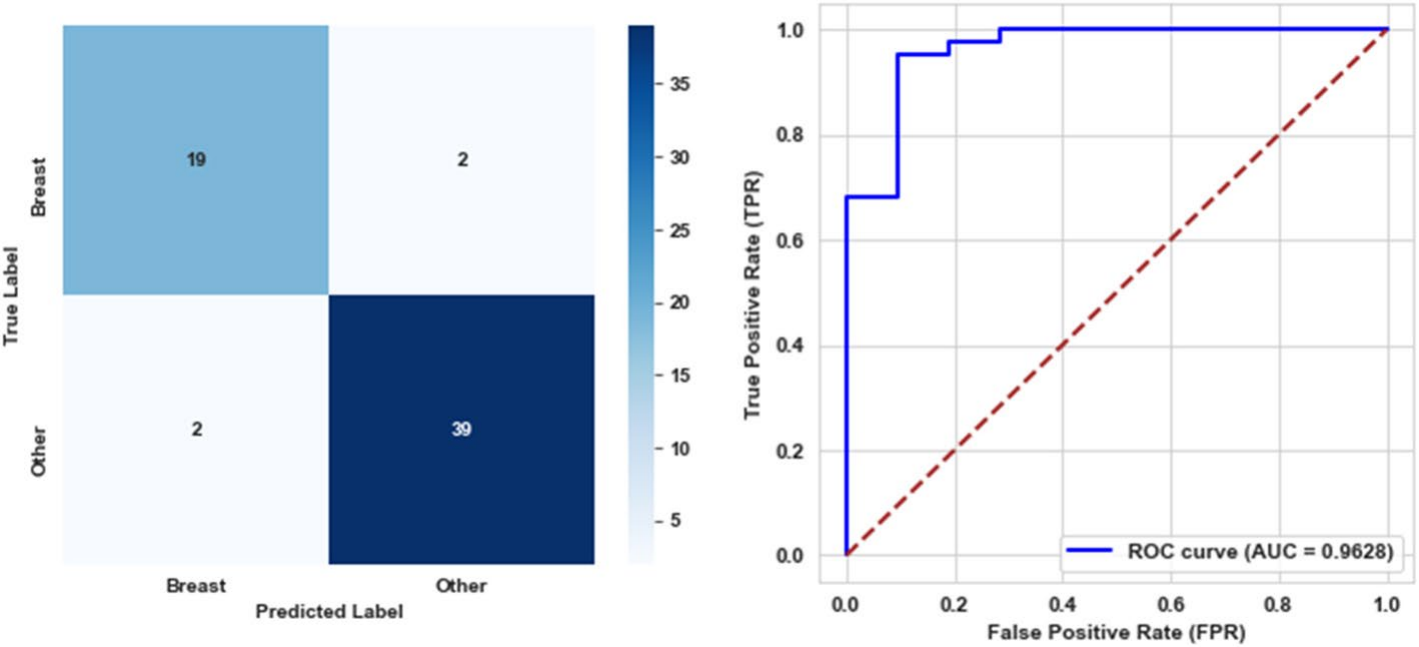
Confusion matrix and ROC curve for the third level model

In the third level, the trained model was tested on the evaluation dataset, with an accuracy of 93.55%, precision, recall, and F1-score, reaching 95.12%. Furthermore, it demonstrated a specificity of 90.48%, a balanced accuracy of 92.80%, and an AUC of 96.28%. These results highlight the model's effectiveness in distinguishing positive and negative instances while maintaining promising overall classification performance. The confusion matrix and AUC curve illustrating these outcomes are depicted in Fig. 9.

**Fig. 10:**
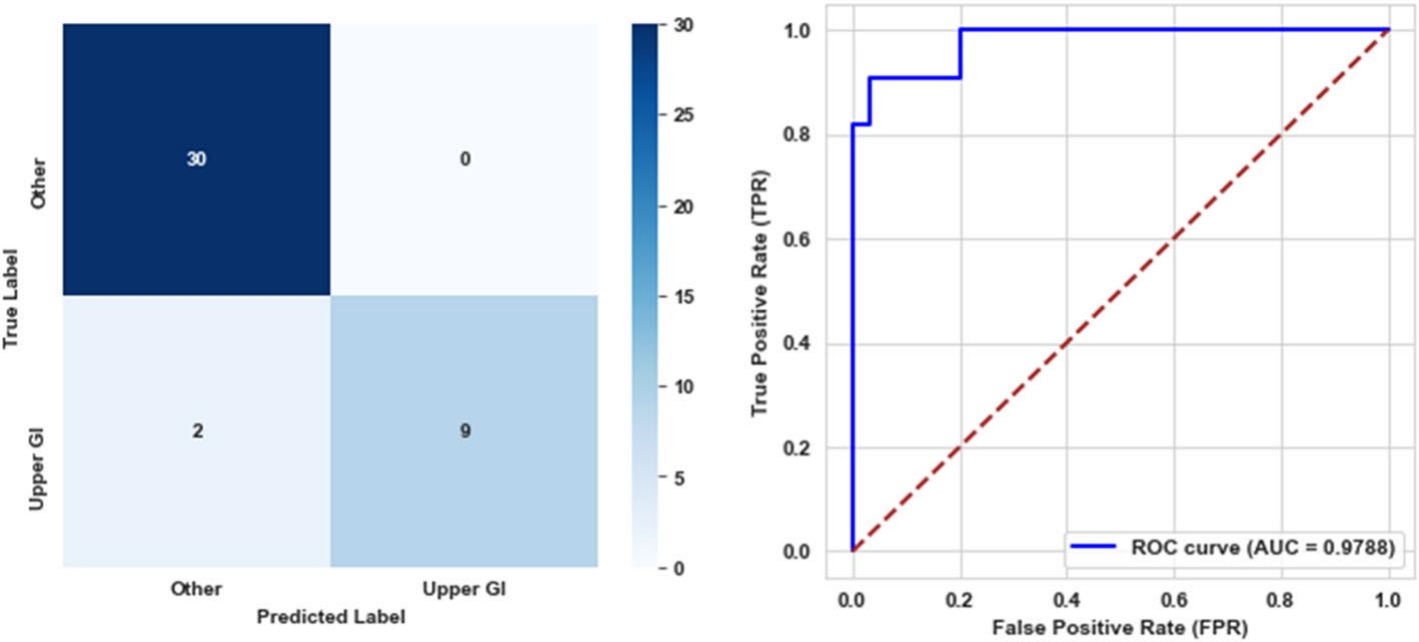
Confusion matrix and ROC curve for the fourth level model

For the fourth level, the trained model was evaluated on the testing dataset, demonstrating an accuracy of 95.12%. The model achieved a precision of 100%, indicating no false positive predictions, while the recall stood at 81.82%. The F1-score reached 90%. Additionally, the model recorded a specificity of 100%, a balanced accuracy of 90.91%, and an AUC of 97.88%, highlighting its promising discriminative power. The corresponding confusion matrix and AUC curve are presented in Fig. 10.

**Fig. 11:**
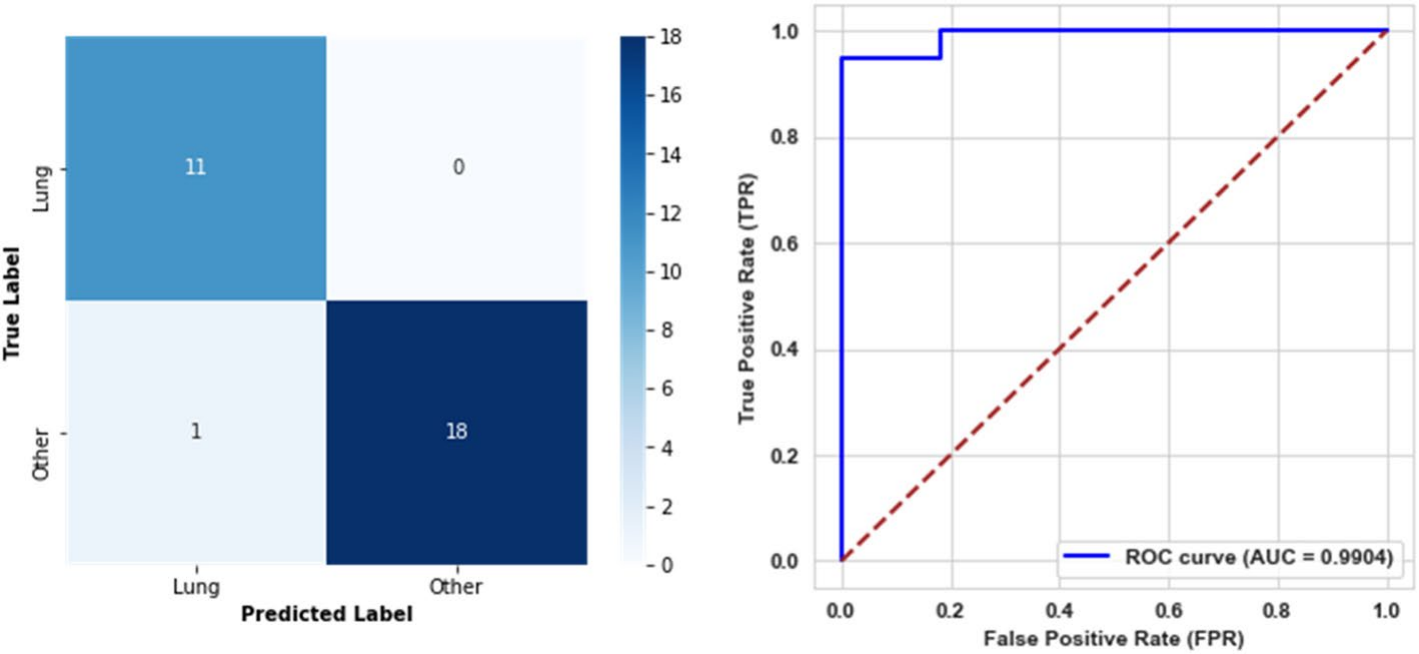
Confusion matrix and ROC curve for the fifth level model

For the fifth level, the model achieved an accuracy of 96.67%, demonstrating a high overall correctness in classification. The precision reached 100%, signifying no false positives, while the recall was 94.74%. The F1-score stood at 97.30%. Furthermore, the model attained a specificity of 100%, a balanced accuracy of 97.37%, and an AUC of 99.04%, emphasizing its capability to distinguish between classes. The confusion matrix and AUC curve are illustrated in Fig. 11.

**Fig. 12:**
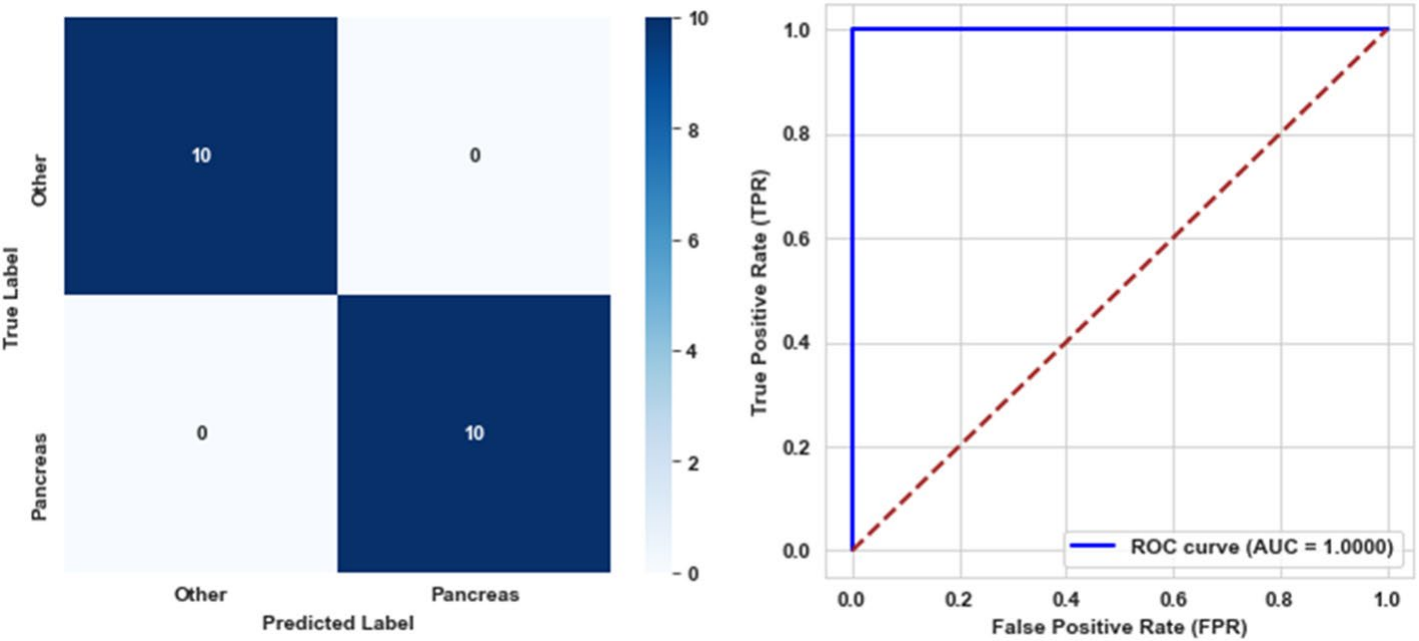
Confusion matrix and ROC curve for the sixth level model

**Fig. 13:**
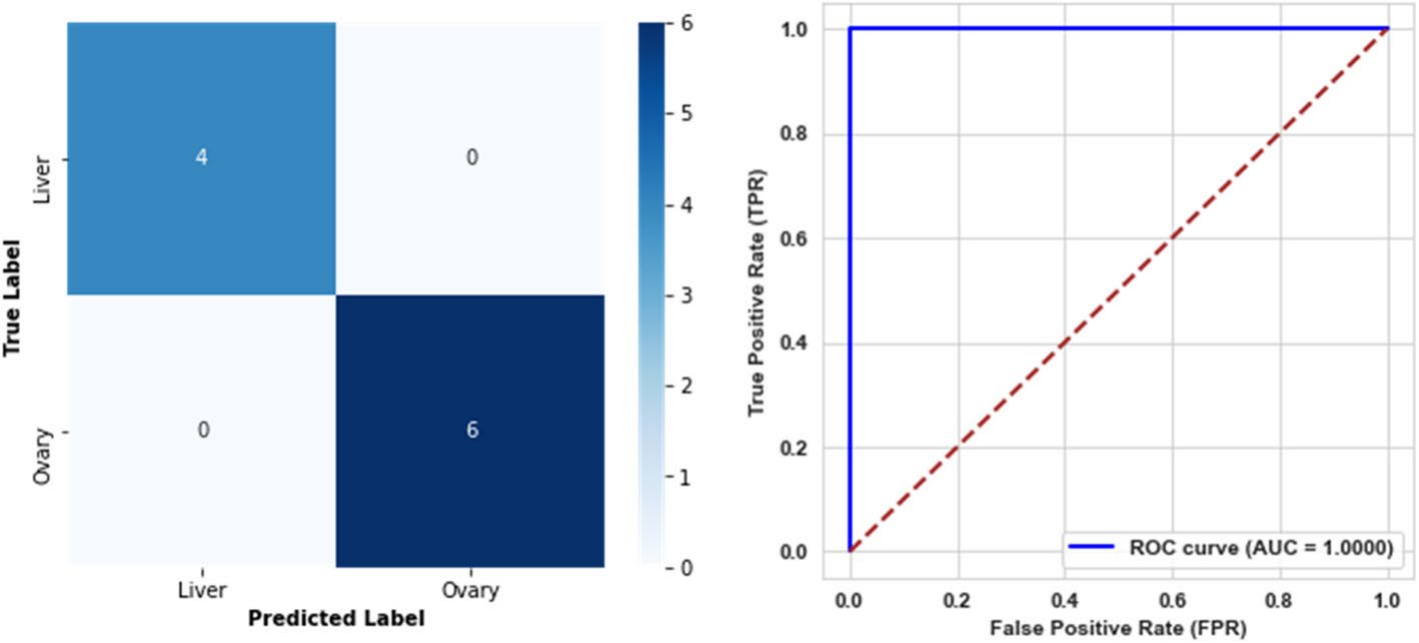
Confusion matrix and ROC curve for the seventh level model

The models achieved perfect classification performance for both the sixth and seventh levels, attaining 100% accuracy, precision, recall, F1-score, specificity, balanced accuracy, and AUC. These results indicate that the models distinguished between classes without any misclassifications. The confusion matrices and AUC curves for both levels are presented in Figs. 12 and 13, respectively.

The overall performance of the proposed system was evaluated by averaging the results across all seven classification levels. The system achieved an average accuracy of 96.21%, precision of 97.74%, recall of 94.8%, F1-score of 96.13%, specificity of 96.08%, balanced accuracy of 95.44%, and an AUC of 98.2%. These results highlight the robustness and effectiveness of the proposed approach.

### Benchmarking results

**Fig. 14:**
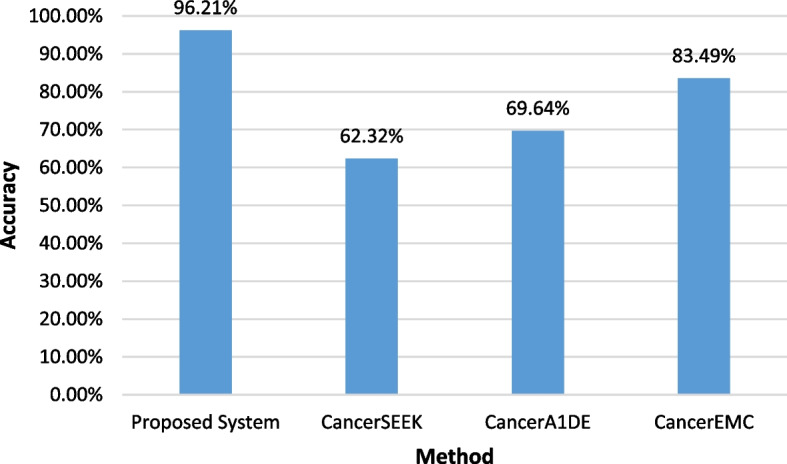
Comparative accuracy analysis of the proposed multi-level cancer classification system and CancerSEEK, CancerA1DE, and CancerEMC

**Fig. 15:**
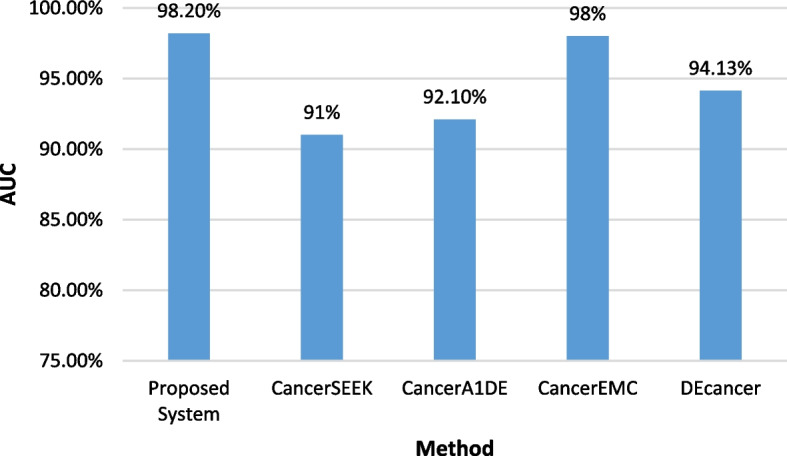
Comparative AUC analysis of the proposed multi-level cancer classification system and CancerSEEK, CancerA1DE, CancerEMC, and DEcancer

The proposed multi-level binary classification system is compared with previously published results using the Cohen et al. dataset. Figure 14 shows that the proposed approach outperforms CancerSEEK, CancerA1DE, and CancerEMC in accuracy measures. Figure 15 shows that the proposed system beats CancerSEEK, CancerA1DE, CancerEMC, and DEcancer in the AUC measure.

The proposed system demonstrates significant advantages over existing methodologies applied to the Cohen et al. dataset. Unlike CancerSEEK, which relies on logistic regression for cancer detection and random forest for cancer-type classification, our approach integrates multiple machine learning models—including XGBoost, Random Forest, Extra Trees, and QDA—allowing for a more comprehensive and adaptable classification process. CancerA1DE, which employs generative learning, may struggle with generalization across different datasets, whereas our system incorporates a robust majority vote feature selection mechanism, ensuring that only the most relevant biomarkers are retained for classification.

While CancerEMC also utilizes ensemble methods such as random forests and bagging classifiers, its interpretability remains a challenge, whereas our approach enhances both interpretability and performance by systematically refining feature selection and classification across multiple levels. Additionally, DEcancer lacks transparency regarding the classifiers and feature selectors, making it difficult to reproduce and validate its results. By adopting a multi-stage binary classification strategy and customizing models for each cancer type, our system achieves promising predictive performance, with an accuracy of 96.21% and an AUC of 98.2%, surpassing previously published results.

## Discussion

This study's stepwise binary classification approach ensures that class distribution remains balanced at each stage, effectively mitigating the risk of severe class imbalance. Unlike methodologies such as CancerEMC, which employ SMOTE to artificially generate synthetic data for balancing, our approach maintains class proportions without introducing synthetic samples. This is crucial for ensuring the robustness and generalizability of the results, as CancerEMC reports SMOTE-based model results where the test data include artificial data that does not necessarily capture the true underlying distribution. Additionally, the training and testing datasets are split approximately stratified, as illustrated in Table 3, ensuring that class distributions in the training and testing sets remain proportionally consistent. This approach preserves the representativeness of the dataset across both phases, thereby enhancing model reliability and reducing the risk of bias that might arise from unbalanced datasets.

Adopting a majority vote feature selection process that integrates multiple selection techniques to identify the most relevant biomarkers for cancer classification. By combining filter, embedded, and wrapper methods, we leverage the strengths of each category to enhance both interpretability and predictive performance. The filter-based techniques, including IV and Chi-Square, assess the statistical relevance of features independently of the model, ensuring that only the most informative variables are retained before training. Meanwhile, embedded methods, such as Random Forest and Extra Tree Feature Importance, utilize tree-based algorithms to rank features based on their contribution to predictive accuracy, providing insights into feature interactions. Finally, the wrapper method, RFE, systematically eliminates less relevant features by iteratively training models on different subsets, optimizing feature selection for improved model performance. L1 Regularization refines the selection process by enforcing sparsity, reducing overfitting, and ensuring that only the most critical biomarkers are retained. By integrating these diverse selection techniques, the proposed system enhances the stability and robustness of feature selection, preventing bias from any single method and ensuring that only the most significant biomarkers contribute to cancer classification.

To justify using three as a threshold for voting, we performed experiments across all seven classification levels using thresholds of 2 and 4 and analyzed the resulting performance metrics and the number of selected features, as reported in Tables 17 and 18. The results show that a threshold of 2, while allowing a broader set of features, led to a decrease in recall (89.22%) and balanced accuracy (91.47%), indicating that including less relevant features introduced noise and reduced classification robustness. On the other hand, a stricter threshold of 4 improved specificity (94.55%) but slightly reduced recall (91.04%) and AUC (96.79%), suggesting that an overly restrictive feature set might exclude critical biomarkers. In contrast, using a threshold of 3, the proposed system achieved an optimal balance across all performance metrics. These results confirm that a threshold of 3 effectively preserves essential features while mitigating overfitting and optimizing classification performance across all levels.

**Table 17:** Performance metrics and number of selected features using a threshold of 2 across all classification levels

Level	#SF	Acc	Precision	Recall	F1	Specificity	BA	AUC
Level 1	28	1	1	1	1	1	1	1
Level 2	27	0.8911	0.9048	0.9194	0.912	0.8462	0.8828	0.94
Level 3	33	0.9355	0.9512	0.9512	0.9512	0.9048	0.928	0.9652
Level 4	31	0.9268	1	0.7273	0.8421	1	0.8636	0.9667
Level 5	31	0.9333	0.9474	0.9474	0.9474	0.9091	0.9282	0.9856
Level 6	32	0.8	0.875	0.7	0.7778	0.9	0.8	0.97
Level 7	28	1	1	1	1	1	1	1
**Avg**	30	0.9267	0.9541	0.8922	0.9186	0.9372	0.9147	0.9754

**Table 18:** Performance metrics and number of selected features using a threshold of 4 across all classification levels

Level	#SF	Acc	Precision	Recall	F1	Specificity	BA	AUC
Level 1	20	0.9945	1	0.9901	0.995	1	1	1
Level 2	21	0.8515	0.873	0.8871	0.88	0.7949	0.841	0.9218
Level 3	17	0.8548	0.9211	0.9211	0.8861	0.8571	0.8554	0.9384
Level 4	18	0.9024	0.8889	0.7273	0.8	0.9667	0.847	0.9394
Level 5	15	0.9667	1	0.9474	0.973	1	0.9737	0.9856
Level 6	17	0.95	1	0.9	0.9474	1	0.95	0.99
Level 7	21	1	1	1	1	1	1	1
**Avg**	18	0.9314	0.9547	0.9104	0.9259	0.9455	0.9239	0.9679

To further validate the effectiveness of the proposed majority vote feature selection approach, we compared its performance against recent metaheuristic optimization-based feature selection algorithms, including Energy Valley Optimization (EVO) [44], Fick's Law Algorithm (FLA) [45], Fox Optimizer (FOX) [46], and Physical Phenomenon of RIME-ice (RIME) [47]. These methods were used as baselines, and their performance was evaluated across all seven classification levels. The results for each method are reported in Tables 19, 20, 21, 22. Among these, EVO achieved an average accuracy of 88.88%, while FLA, FOX, and RIME obtained 88.78%, 88.59%, and 88.75%, respectively. This demonstrates that the hybrid feature selection approach effectively enhances predictive performance by integrating filter, embedded, and wrapper methods to select the most relevant biomarkers, surpassing the optimization-based feature selection techniques in robustness and classification accuracy.

**Table 19:** Performance metrics and number of selected features using EVO across all classification levels

Level	#SF	Acc	Precision	Recall	F1	Specificity	BA	AUC
Level 1	22	0.978	0.9899	0.9703	0.98	0.9877	0.979	0.9978
Level 2	27	0.8218	0.8548	0.8548	0.8548	0.7692	0.812	0.9289
Level 3	21	0.9194	0.9286	0.9512	0.9398	0.8571	0.9042	0.9663
Level 4	15	0.9024	0.8889	0.7273	0.8	0.9667	0.847	0.9515
Level 5	22	0.8	0.8824	0.7895	0.8333	0.8182	0.8038	0.9282
Level 6	17	0.8	0.7143	1	0.8333	0.6	0.8	0.97
Level 7	19	1	1	1	1	1	1	1
**Avg**	20	0.8888	0.8941	0.8990	0.8916	0.8570	0.8780	0.9632

**Table 20:** Performance metrics and number of selected features using FLA across all classification levels

Level	#SF	Acc	Precision	Recall	F1	Specificity	BA	AUC
Level 1	17	0.9835	0.9712	1	0.9854	0.963	0.9815	0.9996
Level 2	23	0.8515	0.8852	0.871	0.878	0.8205	0.8457	0.9264
Level 3	20	0.9194	0.95	0.9268	0.9383	0.9048	0.9158	0.9774
Level 4	19	0.9268	1	0.7273	0.8421	1	0.8636	0.9803
Level 5	16	0.9333	0.9474	0.9474	0.9474	0.9091	0.9282	0.9904
Level 6	27	0.6	0.75	0.3	0.4286	0.9	0.6	0.87
Level 7	24	1	1	1	1	1	1	1
**Avg**	21	0.8878	0.9291	0.8246	0.8600	0.9282	0.8764	0.9634

**Table 21:** Performance metrics and number of selected features using FOX across all classification levels

Level	#SF	Acc	Precision	Recall	F1	Specificity	BA	AUC
Level 1	23	0.978	0.9802	0.9802	0.9802	0.9753	0.9778	0.9947
Level 2	21	0.8416	0.8382	0.9194	0.8769	0.7179	0.8187	0.9264
Level 3	27	0.9194	0.95	0.9268	0.9383	0.9048	0.9158	0.9762
Level 4	18	0.8293	1	0.3636	0.5333	1	0.6818	0.9045
Level 5	21	0.8333	0.8889	0.8421	0.8649	0.8182	0.8301	0.9426
Level 6	22	0.8	0.8	0.8	0.8	0.8	0.8	0.8
Level 7	24	1	1	1	1	1	1	1
**Avg**	22	0.8859	0.9225	0.8332	0.8562	0.8880	0.8606	0.9349

**Table 22:** Performance metrics and number of selected features using RIME across all classification levels

Level	#SF	Acc	Precision	Recall	F1	Specificity	BA	AUC
Level 1	22	0.9945	0.9902	1	0.9951	0.9877	0.9938	0.9999
Level 2	27	0.8119	0.8413	0.8548	0.848	0.7436	0.7992	0.9272
Level 3	17	0.8871	0.9722	0.8537	0.9091	0.9524	0.903	0.9599
Level 4	19	0.9024	0.8889	0.7273	0.8	0.9667	0.847	0.9288
Level 5	25	0.8667	0.8947	0.8947	0.8947	0.8182	0.8565	0.9569
Level 6	17	0.75	0.8571	0.6	0.7059	0.9	0.75	0.93
Level 7	21	1	1	1	1	1	1	1
**Avg**	21	0.8875	0.9206	0.8472	0.8790	0.9098	0.8785	0.9575

To further establish the superiority of the proposed majority voting feature selection approach, we conducted two statistical tests to compare its performance against recent metaheuristic optimization-based feature selection methods, including EVO, FLA, FOX, and RIME. The statistical tests were conducted by comparing the performance of the proposed majority voting feature selection method against baseline optimization-based feature selection methods across the seven classification levels. The results from each level were used to compute the statistical significance of differences in accuracy, F1-score, and balanced accuracy.

First, we performed the Wilcoxon Signed-Rank test [48] to determine whether the observed improvements in accuracy, F1-score, and balanced accuracy were statistically significant. The results indicate that the proposed method consistently outperformed all baseline optimization methods across all three metrics, with a Wilcoxon T value of 0 and a *p*-value of approximately 0.0277, confirming statistical significance at the 0.05 level.

Similarly, the Friedman Rank test [49] was applied to compare the ranking of feature selection methods. The results showed that the proposed majority voting approach achieved the highest ranking across accuracy, F1-score, and balanced accuracy, with a ranking score 33 for each metric. In contrast, EVO, FLA, FOX, and RIME exhibited significantly lower rankings, with scores ranging from 16 to 22.5 depending on the metric, as shown in Table 23.

**Table 23:** Friedman rank results for the majority voting, EVO, FLA, FOX, and RIME feature selection methods

Metric	Majority Voting	EVO	FLA	FOX	RIME
Accuracy	33	16.5	22	17	16.5
F1-score	33	17.5	21.5	16.5	16.5
Balanced Accuracy	33	16	22.5	17	16.5

The Friedman test produced statistically significant results at the 0.05 level for all three metrics, with *p*-values of 0.0065 for accuracy, 0.0097 for F1-score, and 0.0067 for balanced accuracy, demonstrating that the differences in performance among the feature selection methods are not due to random variation. These findings confirm that the proposed majority voting feature selection method improves statistically significantly over recent optimization-based approaches, leading to promising classification performance.

The selection of classification algorithms in the proposed system was guided by a rigorous evaluation of 16 different classifiers, with their performance assessed at each classification level (detailed in Supplementary Information, Online Resource 5), including RF, Gradient Boosting (GB) [50], AdaBoost [51], SVM [52], Logistic Regression (LR) [53], Bagging [54], k-Nearest Neighbors (k-NN) [55], Gaussian Naive Bayes (NB) [56], Decision Trees (DT) [57], Multi-Layer Perceptron (MLP) [58], XGBoost [37], LGBM [59], CatBoost [60], ET [38], QDA [39], and Linear Discriminant Analysis (LDA) [39]. The average performance results of these algorithms are shown in Table 24.

**Table 24:** Average performance results for machine learning algorithms across all classification levels

Classifier	Acc	Precision	Recall	F1	Specificity	BA	AUC
RF	0.92031	0.95000	0.89566	0.91553	0.90842	0.90204	0.97568
GB	0.90750	0.93295	0.88178	0.90220	0.90114	0.89146	0.96994
AdaBoost	0.89268	0.91194	0.86266	0.88315	0.90146	0.88206	0.94112
SVM	0.75789	0.78790	0.78426	0.76688	0.64099	0.71263	0.82048
LR	0.78333	0.84037	0.76148	0.78999	0.74176	0.75162	0.86156
Bagging	0.88583	0.96174	0.80443	0.86577	0.93920	0.87181	0.95320
k-NN	0.72902	0.78637	0.71202	0.70726	0.70397	0.70800	0.79157
NB	0.73088	0.84886	0.66816	0.68250	0.87397	0.77106	0.87497
DT	0.86750	0.90768	0.81626	0.85359	0.91558	0.86592	0.86592
MLP	0.66049	0.65375	0.70442	0.66114	0.57332	0.63887	0.67293
XGBoost	0.91540	0.93671	0.88308	0.90545	0.91213	0.89760	0.96907
LGBM	0.91488	0.93484	0.88426	0.90499	0.90847	0.89636	0.97046
CatBoost	0.91962	0.93310	0.90416	0.91554	0.90176	0.90296	0.97375
ET	0.91709	0.95498	0.89075	0.91755	0.92203	0.90639	0.97746
QDA	0.84050	0.90225	0.79034	0.81101	0.93963	0.86498	0.92333
LDA	0.84630	0.89649	0.81888	0.85161	0.84408	0.83148	0.92607

The decision-making process aimed to maximize predictive performance while maintaining model interpretability and efficiency. At three classification levels—Level 2 (XGBoost and RF), Level 3 (ET and RF), and Level 6 (QDA and XGBoost)—ensemble soft voting was employed, as these classifiers demonstrated the highest individual performance in their respective levels. The combination of these models leverages the strengths of both: XGBoost’s ability to capture complex feature interactions, RF’s robustness against overfitting, ET’s efficiency in handling data, and QDA’s effectiveness in distinguishing class distributions with varying covariance structures.

For the remaining four levels, ensemble soft voting among the top-performing classifiers did not yield significant improvement; thus, a single classifier was selected. At Level 4, ET was chosen as the best-performing model. Also, at Level 5, QDA was selected as the best-performing model. For the first and final classification levels, where multiple classifiers demonstrated comparable performance, the decision was made to avoid introducing additional classification algorithms not already utilized in other levels. This strategy maintained consistency across classification stages and minimized overall system complexity.

The fluctuations in classification performance across the seven levels can be attributed to variations in classification complexity, feature discriminability, and model effectiveness at each stage. The first, sixth, and seventh levels achieve perfect classification, indicating that these cancer types have well-separated feature distributions with highly discriminative biomarkers. However, intermediate levels (2–5) exhibit variations due to the increasing difficulty of distinguishing certain cancer types, where overlapping biomarker distributions make classification more challenging. Level 2 shows the lowest accuracy and specificity due to handling a more ambiguous classification task. Despite these fluctuations, the system maintains promising overall performance, with an average accuracy of 96.21% and an AUC of 98.2%, demonstrating its robustness and effectiveness in multi-stage cancer classification.

To enhance the interpretability of the proposed classification models, SHapley Additive exPlanations (SHAP) [61] was employed as an eXplainable AI (XAI) technique to analyze feature importance across all seven classification levels. SHAP provides a unified measure of feature contribution by estimating the impact of each feature on model predictions, enabling a deeper understanding of the decision-making process. This approach ensures transparency and aids in identifying key biomarkers influencing cancer classification.

**Fig. 16:**
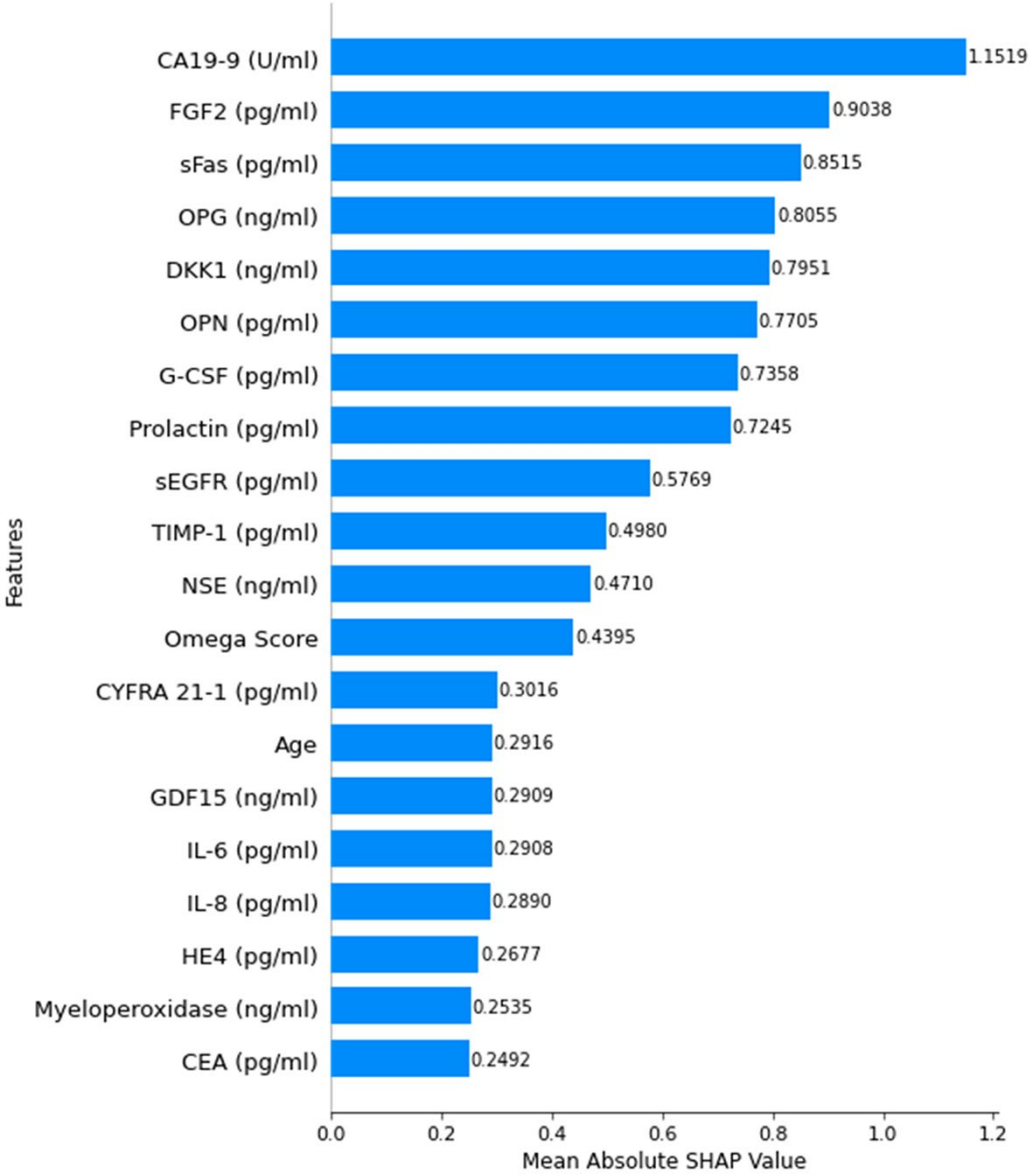
SHAP summary plot for feature importance at classification level 1

Figure 16 shows the SHAP summary for the first classification level, highlighting the most influential features ranked by their mean absolute SHAP values. Notably, CA19-9 exhibits the highest contribution, followed by FGF2, sFas, and OPG, among others. The distribution of SHAP values underscores the relative importance of each biomarker in the classification process at this stage, reinforcing their role in early cancer detection.

**Fig. 17:**
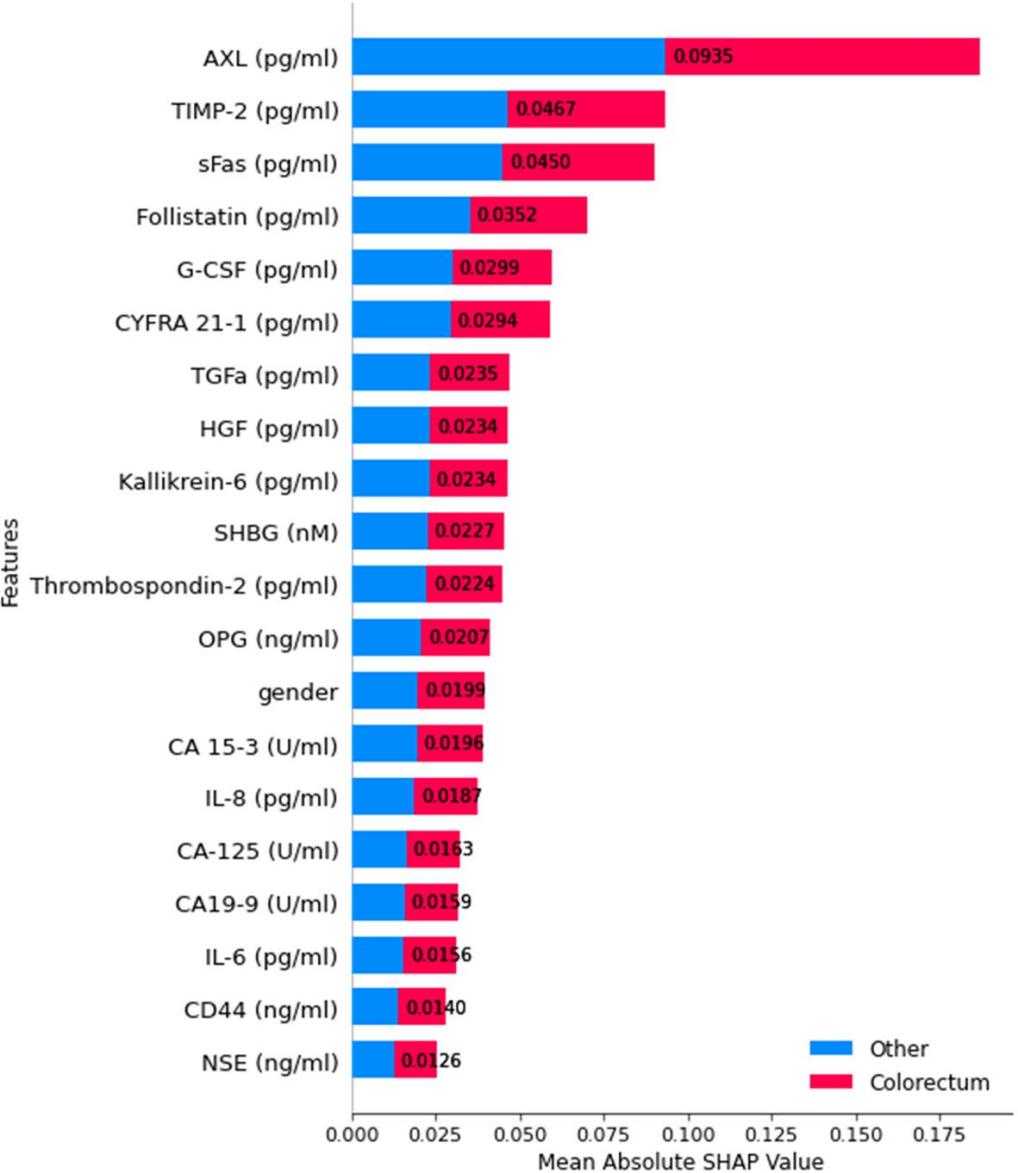
SHAP summary plot for feature importance at classification level 2

For classification level 2, which differentiates between colorectal cancer and other categories, SHAP explainability analysis was performed to identify the most influential features contributing to the model's decision-making. Figure 17 illustrates that AXL exhibited the highest impact on model predictions among the most significant features, followed by TIMP-2, sFas, and Follistatin. These biomarkers are crucial in distinguishing colorectal cancer cases from other categories, suggesting their biological relevance in classification.

**Fig. 18:**
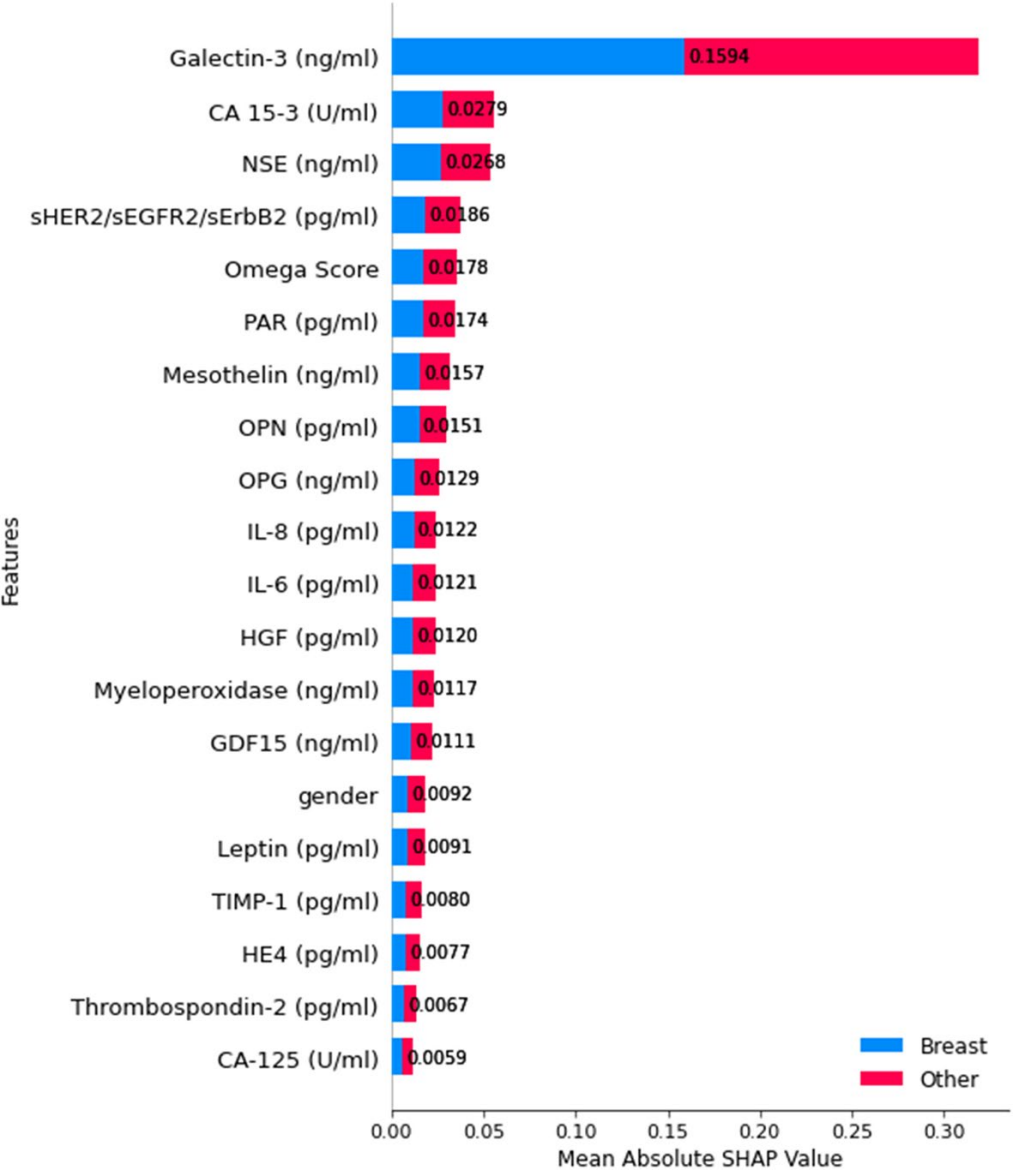
SHAP summary plot for feature importance at classification level 3

Additionally, gender, CA 15–3, and IL-8 contributed to model predictions with relatively lower importance. The color coding further differentiates feature contributions across colorectal and other cancer types, emphasizing how specific features influence predictions differently for each class. This explainability analysis enhances trust in the model's decision-making process and provides insights into the biomarkers most relevant for colorectal cancer classification. For classification level 3, which differentiates breast cancer from other cancer types, Fig. 18 reveals the mean absolute SHAP values for various biomarkers.

Galectin-3 emerged as the most impactful feature, exhibiting the highest influence on model predictions. Additionally, CA 15–3, NSE, and sHER2/sEGFR2/sErbB2 significantly distinguished breast cancer cases. Other biomarkers, such as PAR, Mesothelin, and OPN, contributed to the classification but were of relatively lower importance.

**Fig. 19:**
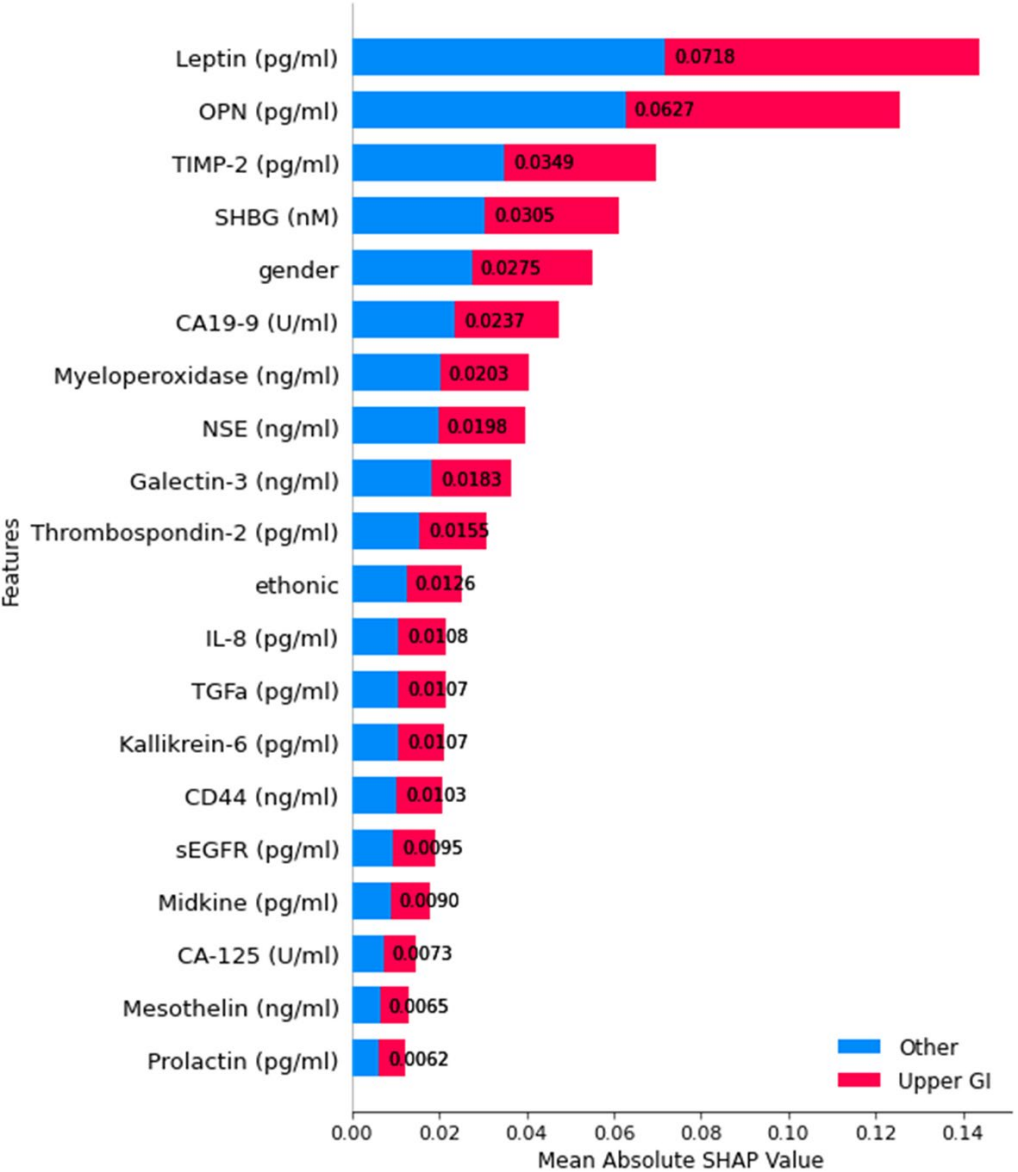
SHAP summary plot for feature importance at classification level 4

For classification level 4, which distinguishes Upper GI cancers from other types, Fig. 19 shows that Leptin and OPN emerged as the two most significant features, demonstrating the highest impact on model predictions. Additionally, TIMP-2, SHBG, and gender were influential in differentiating Upper GI cancers from other malignancies. Other biomarkers, such as CA19-9, Myeloperoxidase, and NSE, contributed to the classification but with comparatively lower importance. This interpretability analysis provides valuable insights into the key factors influencing the Upper GI cancer detection predictive model.

**Fig. 20:**
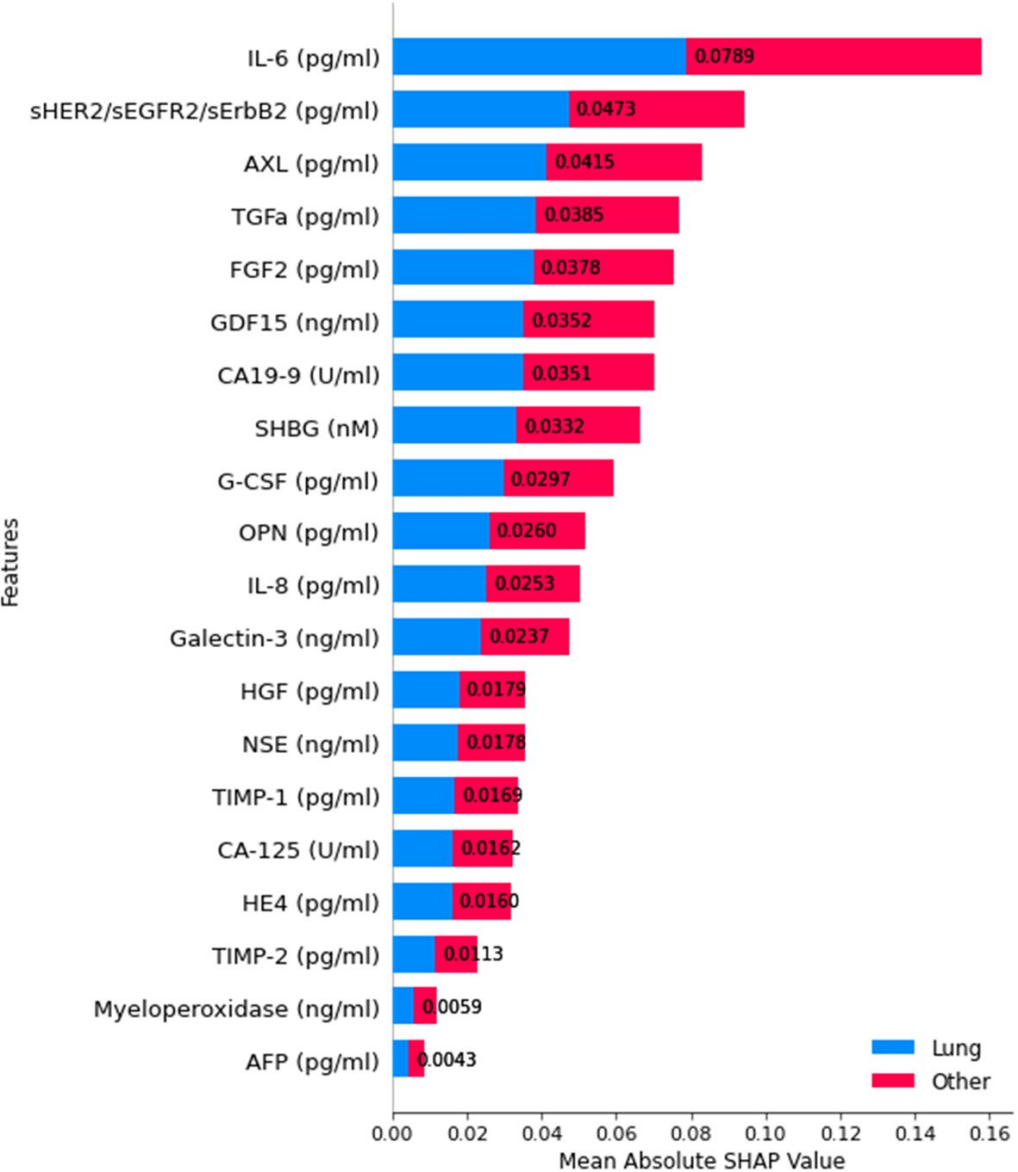
SHAP summary plot for feature importance at classification level 5

For classification level 5, which differentiates Lung cancer from other cancer types, Fig. 20 shows that IL-6 is identified as the most impactful biomarker, playing a crucial role in distinguishing lung cancer from other malignancies. Other highly significant features include sHER2/sEGFR2/sErbB2, AXL, TGFa, and FGF2, demonstrating strong predictive power in lung cancer classification. Additionally, GDF15, CA19-9, SHBG, and G-CSF contribute meaningfully to the model’s decision-making.

**Fig. 21:**
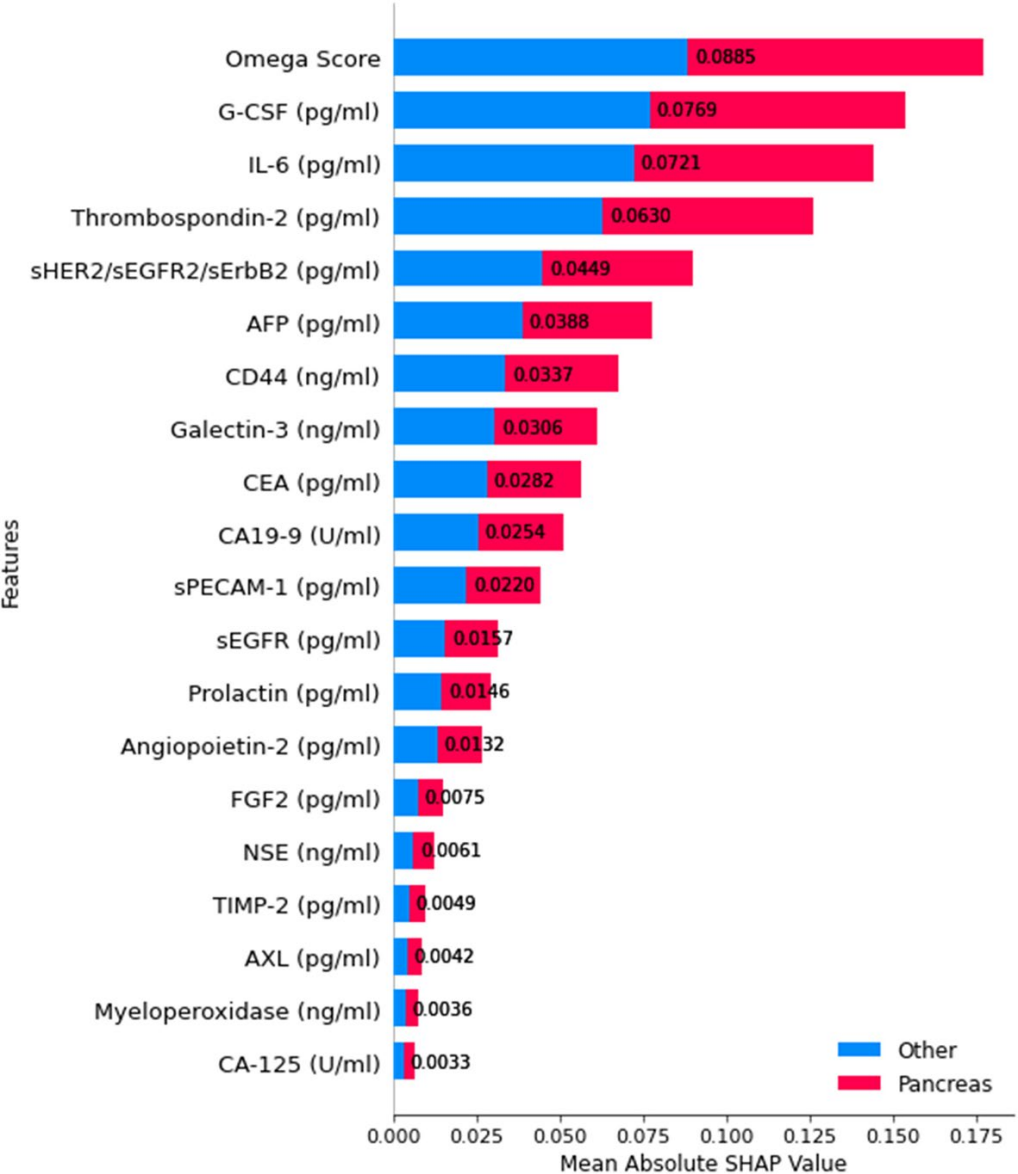
SHAP summary plot for feature importance at classification level 6

For classification level 6, distinguishing Pancreatic cancer from other cancer types, SHAP-based feature importance analysis reveals the most influential biomarkers driving model predictions, as in Fig. 21.

Omega Score emerges as the most significant feature, demonstrating the highest mean absolute SHAP value, suggesting its critical role in distinguishing pancreatic cancer. Other key biomarkers include G-CSF, IL-6, Thrombospondin-2, and sHER2/sEGFR2/sErbB2, contributing significantly to classification decisions. Additionally, biomarkers such as AFP, CD44, Galectin-3, and CEA exhibit notable importance, reinforcing their relevance in pancreatic cancer detection. This analysis underscores the biological significance of these biomarkers in pancreatic cancer diagnosis and contributes to a more transparent machine-learning-driven classification framework.

**Fig. 22:**
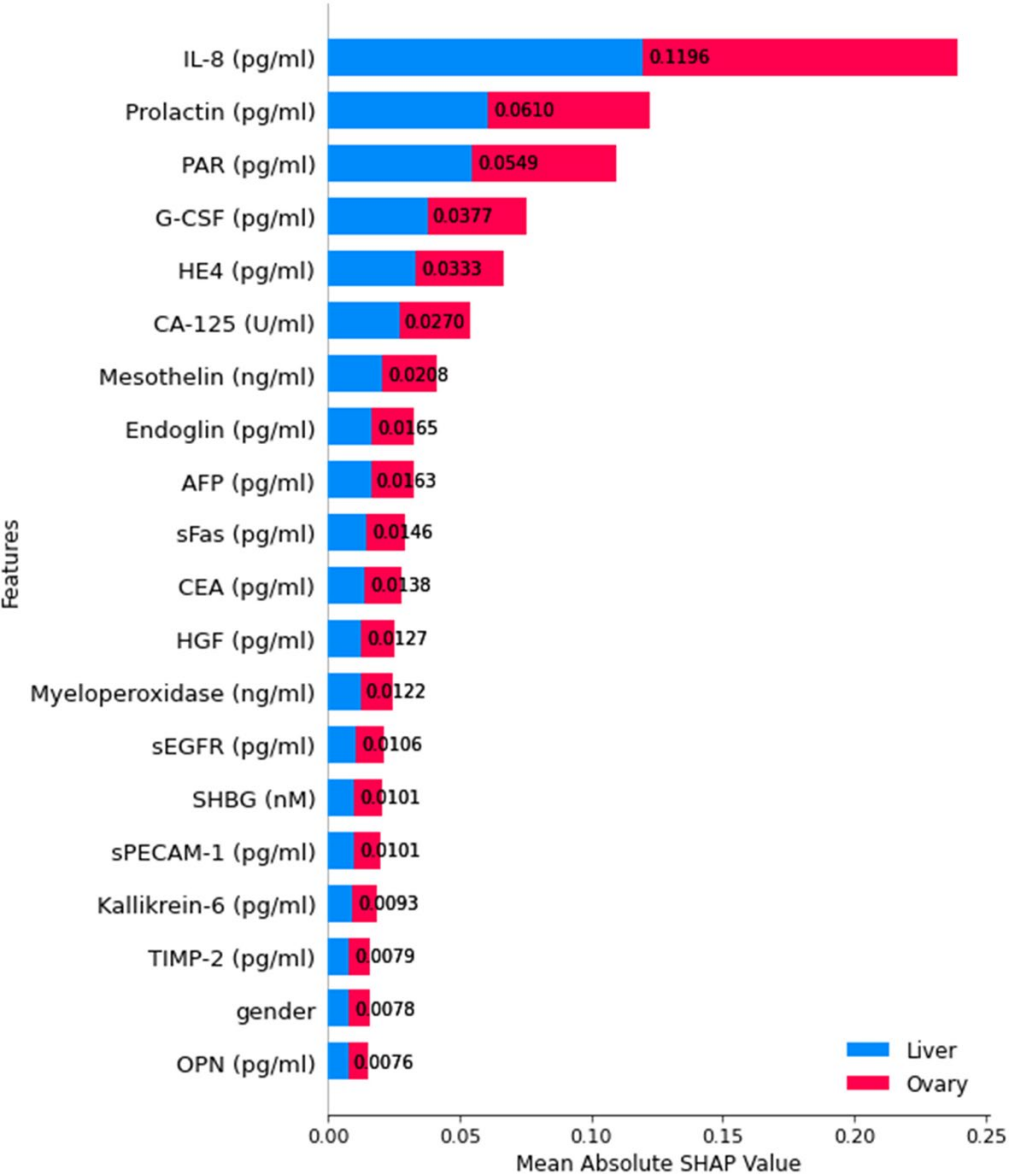
SHAP summary plot for feature importance at classification level 7

For classification level 7, which differentiates Ovarian cancer from Liver cancer, SHAP-based feature importance analysis identifies IL-8 as the most influential biomarker, with the highest mean absolute SHAP value, as shown in Fig. 22.

This suggests that IL-8 plays a crucial role in distinguishing between these two types of cancer. Other significant biomarkers include Prolactin, PAR, G-CSF, HEA, and CA-125. Notably, CA-125 is well-known for its association with ovarian cancer, reinforcing the model’s biological relevance [62]. Additional relevant biomarkers such as Mesothelin, Endoglin, AFP, and CEA contribute to classification, with color-coded SHAP values showing their differential impact between Ovarian and Liver cancer. This feature importance breakdown provides insights into biological markers distinguishing ovarian and liver cancers, reinforcing the robustness of the classification model.

To evaluate the robustness of the proposed model in handling incomplete data, we introduced 10% missingness into the dataset. The missing values were imputed using the k-NN imputer [63], which estimates missing values based on the similarity between data points. The imputed dataset was then processed using the proposed model, and its performance was assessed across multiple evaluation metrics. The results, presented in Table 25, demonstrate that the model maintained high predictive accuracy despite the induced missingness, achieving an average Accuracy of 93.02%, Precision of 95.39%, Recall of 91.08%, F1-score of 93.05%, Specificity of 93.23%, Balanced Accuracy of 92.16%, and AUC of 97.67%.

**Table 25:** Performance evaluation of the proposed model on imputed data with 10% missingness

Level	Acc	Precision	Recall	F1	Specificity	BA	AUC
Level 1	0.989	1	0.9802	0.99	1	0.9901	1
Level 2	0.8515	0.8615	0.9032	0.8819	0.7692	0.8362	0.9231
Level 3	0.9032	0.9268	0.9268	0.9268	0.8571	0.892	0.9605
Level 4	0.9512	1	0.8182	0.9	1	0.9091	0.9879
Level 5	0.9667	1	0.9474	0.973	1	0.9737	0.9856
Level 6	0.85	0.8889	0.8	0.8421	0.9	0.85	0.98
Level 7	1	1	1	1	1	1	1
**Avg**	0.9302	0.9539	0.9108	0.9305	0.9323	0.9216	0.9767

These findings highlight the model's resilience to missing data and confirm that the imputation strategy effectively preserves the underlying data structure without significantly degrading classification performance.

As reported by [64, 65], six commonly utilized biomarkers in clinical practice—AFP, CA 19–9, CA 125, CEA, Prolactin, and CA 15–3—incur an average testing cost of $2 per assay. In contrast, the remaining biomarkers in the dataset are associated with a higher average cost of $5.5 per test, likely reflecting their specialized nature. Furthermore, laboratory protocols indicate that the average processing time for each biomarker test is approximately 2.5 h. Given that the proposed model, across all seven classification levels, leverages all 39 biomarkers in the dataset—consistent with prior studies detailed in Table 1—the estimated total testing clinical lab cost amounts to $193.5, with an approximate time of 97.5 h.

To assess the generalization capability of the proposed model, we attempted to evaluate it on an external dataset. However, to the best of our knowledge, the only publicly available liquid biopsy dataset apart from Cohen et al. is the Hinestrosa et al. [66] sample dataset. Unfortunately, this dataset is highly limited and unsuitable for meaningful validation, as it lacks most of the features in the Cohen et al. dataset. For the first classification level, it includes only 9 out of the 24 required features, rendering it inadequate for evaluation. This limitation highlights a key challenge in our work, emphasizing the need for more extensive and publicly accessible liquid biopsy datasets for robust external validation.

Despite the promising performance of our proposed model, several limitations should be acknowledged. First, the control group in this Cohen et al. dataset was restricted to healthy individuals, whereas in a real-world cancer screening scenario, individuals may present with inflammatory or other non-cancerous conditions. This could potentially lead to a higher false positive rate than observed in our study. Second, our study was limited by the lack of a completely independent external test set. Although cross-validation provides strong internal validation, the absence of an independent dataset for testing prevents us from thoroughly assessing the model's generalizability in real-world clinical settings. Third, the cancer cases in the Cohen et al. dataset were well-defined, with no overlapping or ambiguous diagnoses. However, in practical applications, patients may present with borderline or heterogeneous disease states, which could impact the model’s classification performance.

## Conclusion

This paper presented a comprehensive multi-level classification system for early cancer diagnosis applying ML techniques on liquid biopsy data. Our approach offers a non-invasive method capable of identifying seven distinct cancer types, including colorectal, breast, upper gastrointestinal, lung, pancreas, ovarian, and liver cancer. Each cancer type's dataset was developed using iterative refining through a multi-stage binary classification framework. Additionally, by combining six distinct methods and utilizing a voting technique, the proposed feature selection process aided in identifying essential features crucial for precise predictions. Moreover, tailoring machine learning classifiers to each cancer type enhanced model performance, ensuring reliable and clinically relevant results. The proposed system achieved promising results, with an average accuracy rate of 96.21% and an AUC score of 98.2%, highlighting its potential to enhance clinical diagnostics and patient care. The proposed system contributes to improving patient outcomes and guiding medical interventions by providing early detection capabilities and improving the tissue localization process.

Building upon the current framework, future research could expand the system to detect a broader range of cancer types, particularly rare or understudied malignancies, to enhance its clinical applicability. Integrating longitudinal data from liquid biopsy samples could allow for disease monitoring, facilitating early intervention and personalized treatment strategies. Additionally, incorporating emerging technologies such as single-cell sequencing and spatial transcriptomics could provide deeper insights into tumor heterogeneity, potentially improving the model’s sensitivity and predictive capabilities.

A key avenue for advancement lies in integrating multimodal data, including imaging, genomic, transcriptomic, proteomic, and metabolomic information. Combining these diverse data types could improve the robustness and interpretability of the model, enabling a more comprehensive understanding of cancer biology. For instance, leveraging radiomics features from imaging studies alongside liquid biopsy biomarkers may refine diagnostic accuracy, while integrating metabolomic profiles could help differentiate cancerous from benign conditions, thereby reducing false positives.

Furthermore, external validation remains a crucial next step. To ensure the reliability and generalizability of the identified biomarkers, future work should involve validation using independent datasets. Collaborations with clinical research institutions could facilitate access to diverse patient cohorts, strengthening the model’s real-world applicability. Additionally, investigating the role of these biomarkers across different populations and ethnic groups could enhance the system’s clinical utility by addressing potential demographic biases.

Lastly, as multi-omics data integration techniques evolve, incorporating advanced computational approaches—such as graph neural networks and self-supervised learning—may further improve the system’s ability to uncover hidden relationships between biomarkers. These innovations could pave the way for a next-generation diagnostic platform capable of highly accurate, non-invasive cancer detection and monitoring.

## Supplementary Information

Supplementary Material 1.

Supplementary Material 2.

Supplementary Material 3.

Supplementary Material 4.

Supplementary Material 5.

## Data Availability

The datasets analyzed during the current study are publicly available and can be accessed through the original publisher [https://github.com/SaraEl-Metwally/Towards-Precision-Oncology/tree/main/Original_DataSet] [8]. To ensure reproducibility of the results, the trained models and the dataset used in this study are made publicly available via the GitHub repository [https://github.com/SaraEl-Metwally/Towards-Precision-Oncology].
