# Supplementary material for: Towards precision oncology: a multi-level cancer classification system integrating liquid biopsy and machine learning
Source: BioData Min. 2025 Apr 11;18:29. doi: 10.1186/s13040-025-00439-8 (PMC11987386; doi:10.1186/s13040-025-00439-8)
Supplement: Supplementary file 1 — Supplementary Material 1. [file 13040_2025_439_MOESM1_ESM.docx]

**Towards Precision Oncology: A Multi-Level Cancer Classification System Integrating Liquid Biopsy and Machine Learning**

**Amr Eledkawy, Taher Hamza, and Sara El-Metwally***

*****Address correspondence to this author at the Department of Computer Science, Faculty of Computers and Information, Mansoura University, 35516, Mansoura, Egypt; E-mail: [sarah_almetwally4@mans.edu.eg](mailto:sarah_almetwally4@mans.edu.eg).

**Table S1** Description and Units of Biomarker Features in the Cohen et al. Dataset

| **Input Features** | **Feature Description** | **Unit** |
| --- | --- | --- |
| TGFa | Circulating Transforming Growth Factor | pg/mL |
| HE4 | Circulating Human Epididymis Protein 4 | pg/mL |
| sFas | Circulating soluble Fas Cell Surface Death Receptor | pg/mL |
| Thrombospondin-2 | Circulating Thrombospondin-2 | pg/mL |
| AFP | Circulating Alpha Fetoprotein Precursor | pg/mL |
| G-CSF | Circulating Granulocyte-Colony Stimulating Factor | pg/mL |
| IL-6 | Circulating Interleukin-6 | pg/mL |
| CA-125 | Circulating Cancer Antigen 125 | U/mL |
| sHER2/sEGFR2/sErbB2 | Circulating sHER2/sEGFR2/sErbB2 | pg/mL |
| TIMP-2 | Circulating Tissue Inhibitor of Metalloproteinases 2 | pg/mL |
| CD44 | Circulating CD44 | ng/mL |
| CA19-9 | Circulating Cancer Antigen 19-9 | U/mL |
| IL-8 | Circulating Interleukin-8 | pg/mL |
| CA 15-3 | Circulating Cancer Antigen 15-3 | U/mL |
| HGF | Circulating Hepatocyte Growth Factor | pg/mL |
| OPG | Circulating Osteopontin | ng/mL |
| GDF15 | Circulating Growth Differentiation Factor 15 | ng/mL |
| Leptin | Circulating Leptin Concentration in pg/mL | pg/mL |
| Myeloperoxidase | Circulating Myeloperoxidase | ng/mL |
| Kallikrein-6 | Circulating Kallikrein-6 | pg/mL |
| TIMP-1 | Circulating Tissue Inhibitor of Metalloproteinases 1 | pg/mL |
| Midkine | Circulating Midkine | pg/mL |
| Prolactin | Circulating Prolactin | pg/mL |
| Mesothelin | Circulating Mesothelin | ng/mL |
| Galectin-3 | Circulating Galectin-3 | ng/mL |
| OPN | Circulating Osteopontin | pg/mL |
| NSE | Circulating Neuron-Specific Enolase | ng/mL |
| sEGFR | Circulating Soluble Epidermal Growth Factor Receptor | pg/mL |
| CEA | Circulating Carcinoembryonic Antigen | pg/mL |
| AXL | Circulating AXL Receptor Tyrosine Kinase | pg/mL |
| sPECAM-1 | Circulating Soluble Platelet and Endothelial Cell Adhesion Molecule 1 | pg/mL |
| SHBG | Circulating Sex Hormone-Binding Globulin | nM |
| Angiopoietin-2 | Circulating Angiopoietin-2 | pg/mL |
| DKK1 | Circulating Dickkopf WNT Signaling Pathway Inhibitor 1 | ng/mL |
| CYFRA 21-1 | Circulating Cytokeratin-19 Fragment | pg/mL |
| PAR | Circulating Protease-Activated Receptor | pg/mL |
| Endoglin | Circulating Endoglin | pg/mL |
| FGF2 | Circulating Fibroblast Growth Factor 2 | pg/mL |
| Follistatin | Circulating Follistatin | pg/mL |
